# Arginine regulates inflammation response-induced by *Fowl Adenovirus *serotype 4 via JAK2/STAT3 pathway

**DOI:** 10.1186/s12917-022-03282-9

**Published:** 2022-05-19

**Authors:** Silin Xiang, Ruiling Huang, Qing He, Lihui Xu, Changkang Wang, Quanxi Wang

**Affiliations:** 1 College of Animal Science (College of Bee Science), Fujian Agriculture and Forestry University, Fuzhou, 350002 P.R. China; 2 Fujian Key Laboratory of Traditional Chinese Veterinary Medicine and Animal Health, Fujian Agriculture and Forestry University, Fuzhou, 350002 P.R. China; 3 University Key Laboratory for Integrated Chinese Traditional and Western Veterinary Medicine and Animal Healthcare in Fujian Province, Fujian Agriculture and Forestry Univesity, Fuzhou, 350002 P.R. China

**Keywords:** Arginine, *Fowl adenovirus* serotype 4, Inflammation, JAK2/STAT3 pathway, Transcriptome, Broiler

## Abstract

**Background:**

*Fowl Adenovirus* serotype 4 (FAdV-4) infection causes severe inflammatory response leading to hepatitis-hydropericardium syndrome (HHS) in poultry. As an essential functional amino acid of poultry, arginine plays a critical role in anti-inflammatory and anti-oxidative stress.

**Results:**

In this study, the differential expression genes (DEGs) were screened by transcriptomic techniques, and the DEGs in gene networks of inflammatory response-induced by FAdV-4 in broiler’s liver were analyzed by Kyoto encyclopedia of genes and genomes (KEGG) enrichment. The results showed that the cytokines pathway and JAK/STAT pathway were significantly enriched, in which the DEGs levels of IL-6, IL-1β, IFN-α, JAK and STAT were significantly up-regulated after FAdV-4 infection. It was further verified with real-time fluorescence quantitative polymerase chain reaction (Real-time qPCR) and Western blotting (WB) in vitro and in vivo. The findings demonstrated that FAdV-4 induced inflammatory response and activated JAK2/STAT3 pathway. Furthermore, we investigated whether arginine could alleviate the liver inflammation induced by FAdV-4. After treatment with 1.92% arginine level diet to broilers or 300 μg/mL arginine culture medium to LMH cell line with FAdV-4 infection at the same time, we found that the mRNA levels of IL-6, IL-1β, IFN-α and the protein levels of p-JAK2, p-STAT3 were down-regulated, compared with FAdV-4 infection group. Furthermore, we confirmed that the inflammation induced by FAdV-4 was ameliorated by pre-treatment with JAK inhibitor AG490 in LMH cells, and it was further alleviated in LMH cells treatment with AG490 and ARG.

**Conclusions:**

These above results provide new insight that arginine protects hepatocytes against inflammation induced by FAdV-4 through JAK2/STAT3 signaling pathway.

**Supplementary Information:**

The online version contains supplementary material available at 10.1186/s12917-022-03282-9.

## Introduction

In 1987 *Fowl Adenovirus* serotype 4 (FAdV-4) was firstly reported in Pakistan [1]. Since 2015, FAdV-4 infection had large-scale epidemics in China, which caused incalculable economic losses to the poultry industry [2]. The FAdV-4 infection causes to severe hepatitis-hydropericardium syndrome (HHS) in poultry [3, 4], which pathological features include pericardial effusion, swollen and yellowish livers with bleeding spot and bleeding or enlargement of immune organs [2, 5]. Previous studies demonstrated that the occurrence of the pathological inflammatory response caused by FAdV-4 induced excessive secretion of pro-inflammatory cytokines, resulting in loss of appetite, energy deficiency, and other physiological behaviors and metabolic abnormalities in animals [6]. Moreover, the over-expression of inflammatory factors such as interleukin-1β (IL-1β), interleukin-6 (IL-6) and tumor necrosis factor-alpha (TNF-α) induced by FAdV-4 caused hepatitis and further necrosis [7].

Janus kinase (JAK)/signal transducer and activator of transcription (STAT) signaling pathway widely participates in physiological processes such as inflammation, apoptosis, immunity, and tumor formation [8, 9]. In particular, the JAK2/STAT3 signaling pathway that is activated by inflammatory factors such as IL-6, is most closely related to inflammation [10, 11]. At the initial stage, the activation of JAK2 by inflammatory factors would phosphorylate its downstream STAT3 protein, which ultimately causes the over-expression of cytokines genes and induces cellular pathophysiology [12].

Arginine (ARG) is a conditionally essential amino acid, which participates in vital biological processes, including regulation of the normal function of the immune systems in an organism [13]. Arginine as a dietary nutrient supplement could improve immunologic function, regulate cellular growth, proliferation and immune responses [14]. Our previous studies confirmed that arginine could inhibit FAdV-4 replication in LMH cells [15]. In addition, studies verified that arginine could regulate inflammation of animal. It was indicated that the supplementation of arginine via the jugular vein during early lactation alleviated inflammation of dairy cows [16]. Furthermore, arginine could alleviate the inflammation of excessive immune response [17]. In porcine intestinal epithelial cells (IPEC-J2), L-arginine obviously suppressed the levels of IL-6, IL-8, IL-1β, and TNF-α induced by LPS, alleviated inflammatory response and maintained intestinal integrity [18]. However, it is unknown whether arginine can alleviate the inflammation response induced by FAdV-4.

In this study, we performed transcriptomic technology to screen for DEGs in the liver (the main target organ of FAdV-4) of broilers infected with FAdV-4, and used KEGG to analyze the inflammatory pathway. Meanwhile, qPCR and Western blotting were used to validate inflammation-related factors and pathways in vivo and vitro. Furthermore, the regulation of arginine on cytokines and the JAK2/STAT3 pathway in the inflammatory state was determined.

## Materials and methods

### Virus

FAdV-4 strain NP (TCID_50_ = 10^-6.23^/0.01 mL) was proliferated in DF-1 cell line with RPMI-1640 medium (12,633,012, GiBco, Invitrogen Corp., California, U.S.A), 10% fetal bovine serum (FBS) (Serana, Washington, U.S.A), and 1% penicillin/ streptomycin (10,378,016, GiBco, Invitrogen Corp., California, U.S.A) in a 37 °C incubator (Panasonic, Osaka, Japan) with 5% carbon dioxide (CO_2_).

### Cell culture and treatment

Chicken hepatocellular carcinoma cell line (LMH), a classical hepatocyte model for studying chicken pathogenicity or gene function, was used as a cell model in this experiment [19, 20]. LMH cell line (lot no. 601411-714SF, CLS, Germany) was subculture in modified RPMI-1640 medium (12,633,012, GiBco, Invitrogen Corp., California, U.S.A) with 10% fetal bovine serum (FBS) (Serana, Washington, U.S.A) and 1% penicillin/streptomycin (10,378,016, GiBco, Invitrogen Corp., California, U.S.A) in a 37 °C incubator (Panasonic, Osaka, Japan) with 5% CO_2_. LMHs were treated with arginine at concentrations of 300 μg/mL, while the cells in the control group were cultured in a normal medium for 24 h. The final culture system was 5 mL. Then, cells were collected after infection with 0.5 mL FAdV-4 ([TCID_50_] = 10^–6.23^/0.01 mL) for 2 h. Furthermore, AG490 (a JAK2 inhibitor) was dissolved as 50 nmol/L using dimethyl sulfoxide (DMSO) (D2650, Sigma, Burlington, U.S.A) to treat LMH cells for 2 h at 37 °C [21].

### Animals and treatments

Eighteen 21-day-old normal white-feathered male broilers (Ross 308) were purchased from Fujian Shengnong Food Co. Ltd., China. All birds with negative FAdV-4 antigen and antibody were randomly divided into two groups. Chickens in the infection group were intramuscularly injected with 0.5 mL FAdV4 (TCID_50_ = 10^−6.23^/0.1 mL). Conversely, broilers in the mock group were injected with the same volume of sterile saline. Two days after FAdV-4 infection, liver samples were collected for transcriptome sequencing.

Additionally, 180 twenty-one-day-old broilers (Fujian Shengnong Food Co. Ltd., China) were randomly divided into 3 treatments with 5 replicates per group and 12 broilers per replicate. The FAdV-4 and ARG + FAdV-4 groups were treated by randomly selecting 6 sample broilers of similar weight from the control feeding group and the 1.92% level of arginine feeding group for intramuscular injection of 0.5 mL FAdV-4, respectively. In contrast, 6 broilers from the control feeding group were injected with sterile saline as the mock group. After 2 d, the chickens were deeply anaesthetized with CO_2_ and euthanized for liver collection.

All broilers were fed with corn-soybean meal diet. The diets were formulated to meet the nutritional requirements of broilers as defined by the National Research Council of China (NRC) [22, 23]. The composition and nutritional components of broiler diets are shown in Table 1. Our previous results showed that the 1.92% arginine nutrient level had the most significant effect on resistance to FAdV-4 infection in broilers. Thus, the ARG treatment was defined as 1.92% of the total nutrient level [24].

**Table 1:** Composition and nutrient levels of white-feathered broiler diets (air-dry basis)

	Arginine levels in diet (%)	
	1.20%	1.92%
Ingredients		
Corn	62.78	64.67
Corn gluten meal	16.62	14.42
Soybean meal	9.36	10.47
Puffed soybean	3.00	3.00
Limestone	1.25	1.25
CaHPO_4_	2.20	2.20
NaCl	0.30	0.30
L-Lys	0.65	0.65
DL-Met	0.19	0.19
L-Thr	0.09	0.09
L-Trp	0.04	0.04
Zeolite powder	2.28	0.76
Premix^a^	1.00	1.00
ARG	0.24	0.96
Total	100	100
Nutrient levels		
ME(KJ/kg)	12.54	12.54
CP	21.50	21.50
Ca	1.00	1.00
P	0.45	0.45
Lys	1.15	1.15
Met + Cys	0.91	0.91
Thr	0.81	0.81
Trp	0.21	0.21
ARG	1.20	1.92

^a^The premix provides following for per kg diet: vitamin A, 9,800 U; vitamin D_3_, 2,000 U; vitamin E, 21 mg; vitamin K_3_, 2.24 mg; vitamin B_1_, 2.4 mg; vitamin B_2_, 6.1 mg; vitamin B_3_, 36.5 mg; vitamin B_5_, 10.1 mg; vitamin B_6_, 9.8 mg; vitamin B_12_, 0.02 mg; biotin, 0.2 mg; folic acid, 1.12 mg; choline, 1,300 mg; Cu, 8 mg; Fe, 100 mg; Mn, 120 mg; Zn, 100 mg; Se, 0.3 mg; I, 0.7 mg

### KEGG enrichment analysis

Total RNA was extracted from the livers of broilers in FAdV-4 infection group and control group. The appropriate fragments were selected for PCR amplification by agarose gel electrophoresis. Sequencing libraries were constructed and sequenced using Illumina® HiSeq TM 2000 (Illumina, Shenzhen, China). And the quality and saturation of sequenced reads were evaluated. Then,low-quality reads and impurities from the raw reads were removed to obtain clean reads. The RPKM (reads per kilobase per million mapped reads) method was used to screen and analyze DEGs. The *P*-value was controlled by the false discovery rate, while the false discovery rate for DEGs was ≤ 0.001, and its difference ratio was less than twice [2].

The KEGG functional enrichment analysis was used to screen significant pathways. A pathway with *Q* ≤ 0.05 was characterized as enriched. Significant pathway enrichment enabled the identification of biochemical metabolic pathways and signaling pathways associated with DEGs [2].

### Polymerase chain reaction (PCR) detection

The specific primers for FAdV-4 Fiber 1 (Gene ID: 10,399,487) were designed (Table 2) via Primer 5 (Molecular Biology Insights, Cascade, Co). The virus nucleic acid was extracted with the virus DNA extraction kit ((TransGen Biotech, Beijing, China) and amplified by PCR [2]. The PCR reaction system (25 μL) comprised 1 μL of DNA template, 12.5 μL of PCR Nucleotide Mix (10 mmol/L), 9.5 μL of nuclease-free water, and 1 μL of each upstream and downstream primers. The reaction conditions were as follows: denaturation at 95 °C for 30 s, annealing at 55 °C for 30 s, and extension at 72 °C for 30 s, 30 cycles. PCR products were analyzed by 1% agarose gel electrophoresis.

**Table 2:** Primer sequences for PCR

Genes	Sequence (5’to3’)	Product size, bp	GenBank No
FAdV-4 Fiber 1	F: CCCTCGAGATGTCGGCCCTAATCGCCTCC	1315	APA_19531
	R: CGGAATTCGGGGCCCGGAGCATTGT		
β-actin	F: CCCACACCCCTGTGATGAAA	148	NM_205518
	R: TAGAACTTTGGGGGCGTTCG		

### The mRNA levels were determined by qPCR

Total RNA was extracted from chicken livers and LMH cells using TransZol Up RNA Kit (ER501-01, Beijing TranGen Biotech Co. Ltd., China). The concentration and purity of RNA were identified by measuring OD_260_/OD_280_ values with the NanoDrop 2000 spectrophotometer (Thermo Scientific, Waltham, M.A), then they were frozen at -80 °C. The reverse transcription system was as follows: 1 μg of total RNA, 2 μL RT Master Mix (5 ×), and RNase-free water to fill a total 10 μL. The products (cDNA) were stored at -20 °C for subsequent real-time qPCR [25].

We found the gene sequences of chicken β-actin, IL-6, IL-1β, and IFN-α in GenBank with accession numbers of NM_205518, NM_204628, NM_204524, and NM_205427, respectively. Specific primers (Table 3) for qPCR were designed using Primer 15 software. Real-time qPCR was performed to detect the expression levels of IL-6, IL-1β, and IFN-α genes in different cDNA samples using TransStart® Green qPCR SuperMix (AQ101-01, Beijing TranGen Biotech Co. Ltd., China). Refer to Wang [26], the reaction mixtures were incubated in a LightCycler 480 II real-time PCR system (Basel, Switzerland). The relative mRNA expression levels were calculated using the 2^−ΔΔCt^ method, and the data were calibrated as the relative value to the control group. All samples were analyzed in triplicate.

**Table 3:** Primer sequences for real-time qPCR

Genes	Sequence (5’to3’)	Product size, bp	GenBank No
IL-6	F: AAATCCCTCCTCGCCAATCT	133	NM_204628
	R: CCCTCACGGTCTTCTCCATAAA		
IL-1β	F: CTGCCTGCAGAAGAAGCCT	164	NM_204524
	R: TGTCAGCAAAGTCCCTGCTC		
IFN-α	F: CAACGACACCATCCTGGACA	147	NM_205427
	R: ATCCGGTTGAGGAGGCTTTG		
β-actin	F: CTGGCACCTAGCACAATGAA	90	NM_205518
	R: CTGCTTGCTGATCCACATCT		

### Western blotting examines protein levels

Radio immunoprecipitation assay (RIPA) buffer (P0013B, Beyotime, Shanghai, China) encompassing 1% protease (ST506, Beyotime, Shanghai, China) and 1% phosphatase inhibitors (P1045, Beyotime, Shanghai, China) were added to obtain the total protein supernatant. Proteins were detached using 10% sodium dodecyl sulfate–polyacrylamide gel electrophoresis (SDS-PAGE) (P0012A, Beyotime, Shanghai, China) and then were transferred to polyvinylidene fluoride (PVDF) membranes (BSP0161, Pall, New York, U.S.A). After blocking the membranes with 5% skimmed milk (P0216, Beyotime, Shanghai, China), the membranes were transferred to anti-JAK2 (1:1,000; 3230S, Cell Signaling Technology, Boston, U.S.A), anti-STAT3 (1:1,000; 12640S, Cell Signaling Technology, Boston, U.S.A), anti-phospho-JAK2 (1:1,000; 3771S, Cell Signaling Technology, Boston, U.S.A), anti-phospho-STAT3 (1:1,000; 9145S, Cell Signaling Technology, Boston, U.S.A) and anti-β-actin (1:2000; 4970S, Cell Signaling Technology, Boston, U.S.A) at 4 °C overnight [26, 27]. Membranes were washed 3 times with 1 × tris buffered saline tween (TBST) (T1081, Solarbio, Beijing, China), then were incubated with secondary antibody (1:3000; 7074S, Cell Signaling Technology, Boston, U.S.A) that was conjugated with horseradish peroxidase (HRP). Immunoblots were developed using enhanced chemiluminescence (ECL) reaction (KF001, Affinity Biosciences, OH, U.S.A) and were imaged on a ChemiDoc XRS System (BioRad, California, U.S.A). The value of the target proteins were quantitatively calculated using Image J software.

### Statistical analysis

The data were presented as the mean (*n* = 6) ± standard difference (SD) unless noted otherwise. Data were analyzed by independent-samples ANOVA using the SPSS statistical software (Ver.16.0 for windows, SPSS Inc., Chicago, U.S.A). *P* < *0.05* was considered statistically significant. Data handling and statistical processing were performed using GraphPad Prism 7.0 (GraphPad Software, San Diego, CA, U.S.A).

## Results

### FAdV-4 promoted inflammatory cytokines and JAK2/STAT3 signaling pathway in LMH cells

**Fig. 1:**
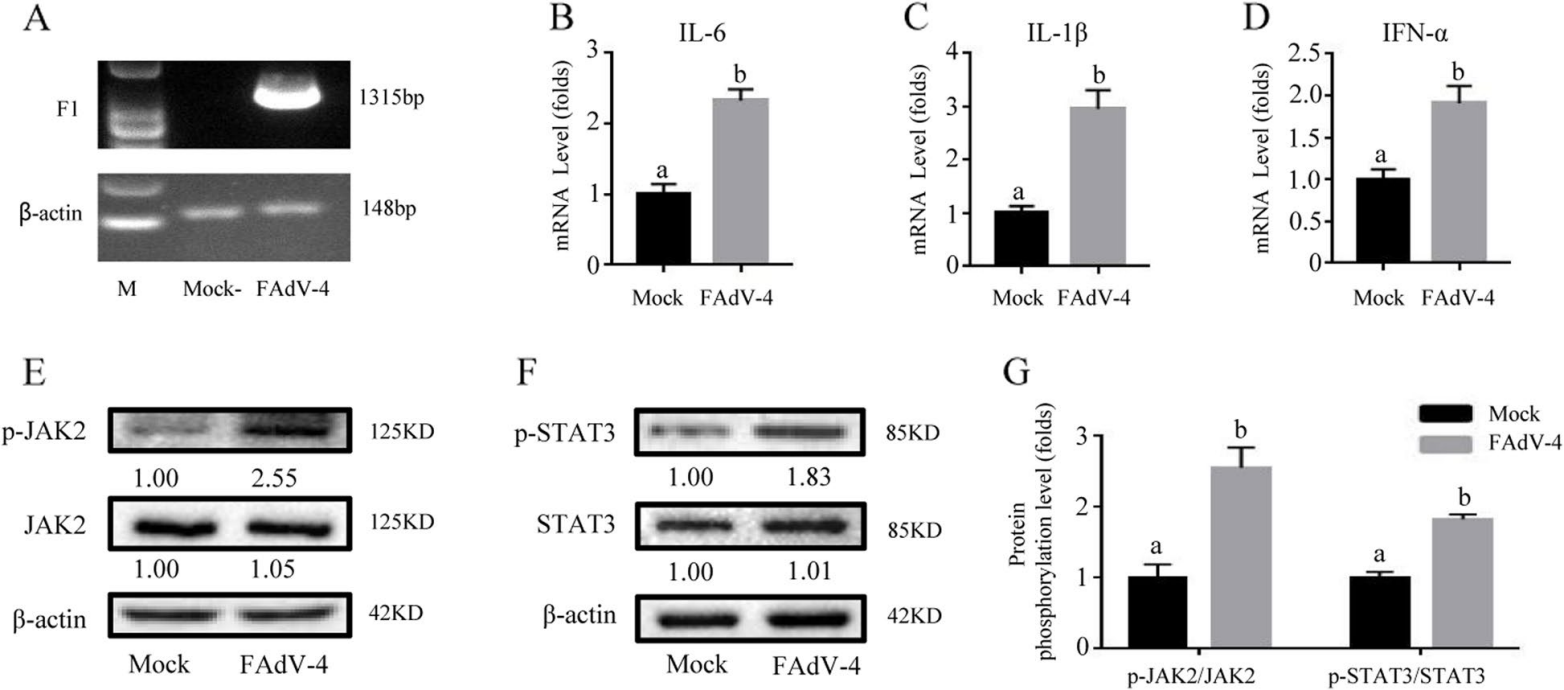
FAdV-4 promoted inflammatory cytokines and the JAK2/STAT3 pathway in LMH cells. The inflammatory indexes of cells infected with or without FAdV-4 were evaluated. After infection with FAdV-4 24 h (**A**), the virus load of FAdV-4 was determined by PCR with the primers of F1 gene, and the β-actin was used as a conference gene. The mRNA levels of cytokines **B** *IL-6*, **C** *IL-1β*, and **D** *IFN-α* was determined by qPCR, while the phosphorylation of crucial proteins (p-JAK2 and p-STAT3) in the JAK2/STAT3 pathway was analyzed by WB (**E**). **G** The Western blotting was analyzed by gray scale and described by histogram, histogram is the result of the ratio of the phosphorylated protein to the total protein, and is the digital embodiment of the protein phosphorylation level of JAK2 and STAT3.Significance between the treatments was determined by T-test analysis using SPSS software (Version 20.0). Means with different alphabets (a, b) denotes significance at *p* < 0.05. β-actin was used as a control for protein loading, and the following were consistent. All values are expressed as Means ± SD (*n* = 6). The a, b, c, d bars in each panel without a common superscript letter were significantly different (*P* < *0.05*). All remain consistent below unless otherwise stated

Results showed that FAdV-4 could significantly induce the mRNA expression levels of IL-6, IL-1β and IFN-α in LMH cells (Fig. 1 A-D) (*P* < *0.05*). Furthermore, the imprinting degree of p-JAK2 and p-STAT3 protein bands in LMH cells infected with FAdV-4 was higher than that in the control group (Fig. 1 E–F). Meanwhile, by analyzing the gray value of the protein bands and transforming them into histograms, we could intuitively observe that the phosphorylation of JAK2 and STAT3 proteins was aggravated by FAdV-4 infection (Fig. 1 G), which indicated that FAdV-4 infection promoted the activation of JAK2 and STAT3.

### Arginine relieved inflammatory factors and JAK2/STAT3 signaling pathway in LMH cells-infected by FAdV-4

**Fig. 2:**
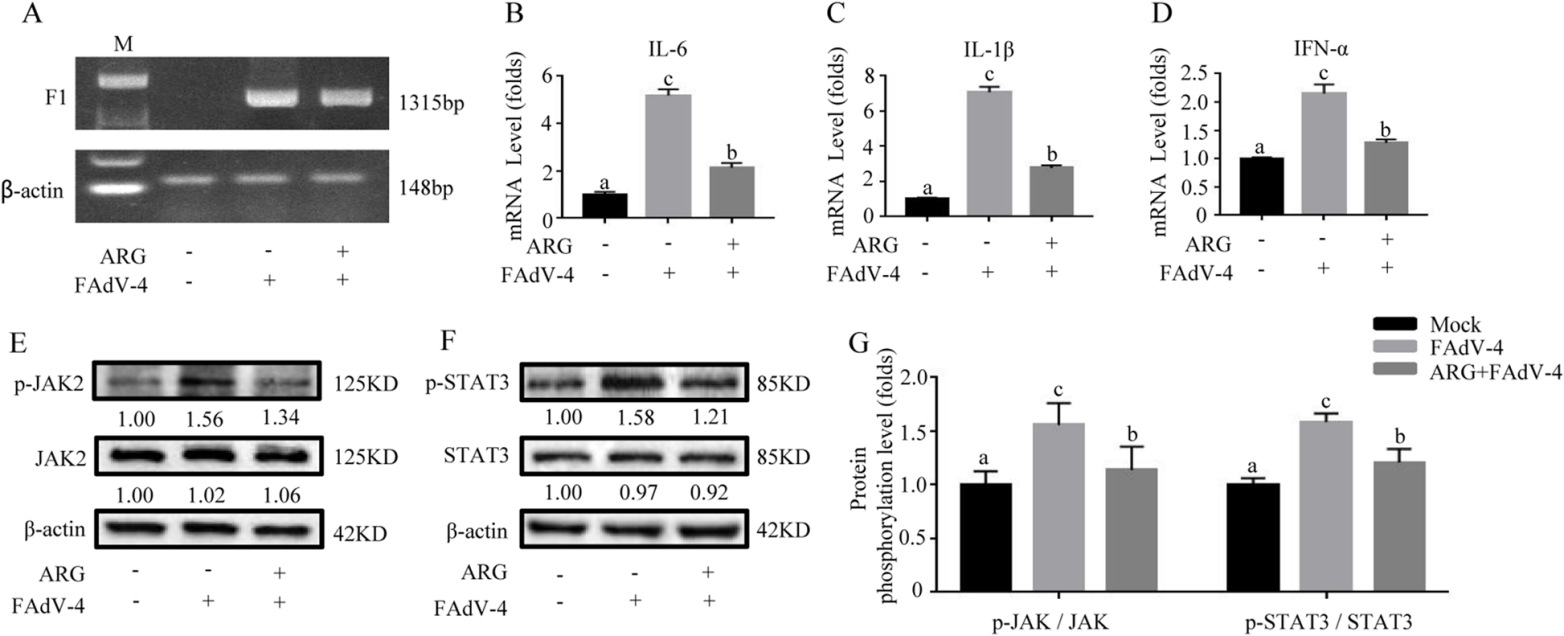
Arginine treatment down-regulated inflammatory response and JAK2/STAT3 pathway-activated by FAdV-4 in LMH cells. The cells were divided into three groups: mock and FAdV-4 groups were cultured with a basal medium containing 10% FBS; ARG + FAdV-4 group was cultured with 300 μg/mL arginine medium. In addition, the FAdV-4 and the ARG + FAdV-4 groups were treated with the FAdV-4 strain NP for 2 h. On the contrary, the mock group was still cultured with a basic medium. After infection with FAdV-4 24 h (**A**), the virus load of FAdV-4 was determined by PCR with the primers of F1 gene, and the β-actin was used as a conference gene. The hepatic mRNA levels of **B** *IL-6*, **C** *IL-1β*, and **D** *IFN-α* were assessed. **E** Similarly, the effect of arginine on the JAK2/STAT3 signaling pathway was determined by evaluating whether arginine interferes with the phosphorylated expression of JAK2 and STAT3. **G** The degree of protein expression was determined by the depth of WB bands. Histogram is the result of the ratio of the phosphorylated protein to the total protein, and is the digital embodiment of the protein phosphorylation level of JAK2 and STAT3.Significance between the treatments was determined by T-test analysis using SPSS software (Version 20.0). Means with different alphabets (a, b) denotes significance at *p* < 0.05

In the present study, the mRNA levels of IL-6, IL-1β and IFN-α in LMH cells -infected with FAdV-4 co-treatment with 300 μg/mL arginine medium were significantly relieved (Fig. 2 A-D) (*P* < *0.05*), compared with FAdV-4 infection cells. Meanwhile, cells-treated with 300 μg/mL arginine medium ruduced the phosphorylation and gray values of p-JAK2 and p-STAT3 after infection with FAdV-4, compared with FAdV-4 infection cells (Fig. 2 E–G) (*P* < *0.05*). These results demonstrated that arginine treatment also could alleviate the inflammatory response of LMH cells infected with FAdV-4.

### JAK2 inhibitor AG490 further alleviates inflammation in FAdV-4-infected LMH cells co-treatment with arginine

**Fig. 3:**
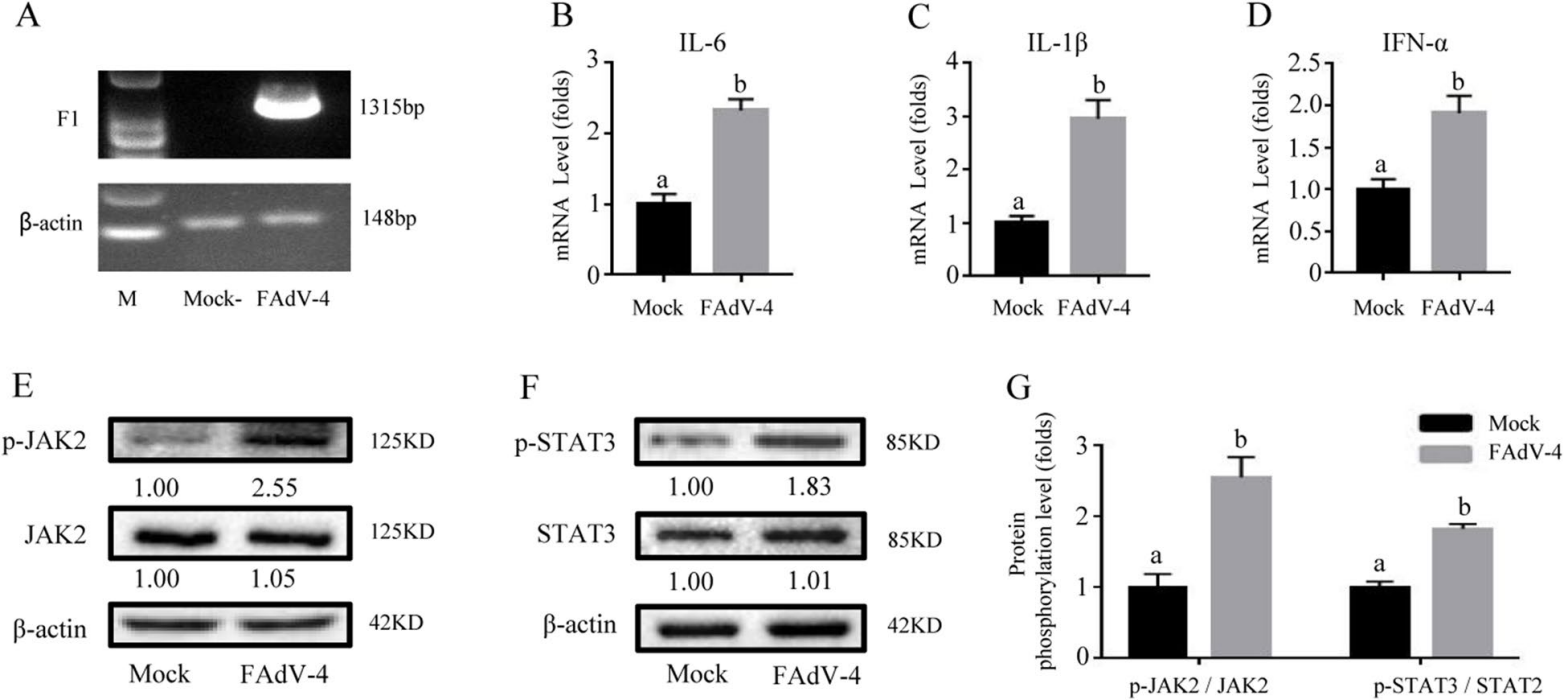
AG490 inhibited the inflammatory effect induced by FAdV-4. To elucidate whether JAK2/STAT3 signaling pathway mediates the effect of arginine in alleviating FAdV-4-induced inflammation, we used AG490, a specific inhibitor of JAK2, in the following trial. LMH cells were treated with 300 μg/mL arginine or 50 nmol/L AG490 and then infected with FAdV-4. After infection with FAdV-4 24 h (**A**), the virus load of FAdV-4 was determined by PCR with the primers of F1 gene, and the β-actin was used as a conference gene. The mRNA levels of **B** *IL-6*, **C** *IL-1β*, and **D** *IFN-α* were measured by real time-qPCR; **E** Protein levels of p-JAK2 and p-STAT3 in LMH cells were analyzed by Western blotting and described by histogram, the degree of protein expression was determined by the depth of WB bands. Histogram is the result of the ratio of the phosphorylated protein to the total protein (**G**), and is the digital embodiment of the protein phosphorylation level of JAK2 and STAT3.Significance between the treatments was determined by T-test analysis using SPSS software (Version 20.0). Means with different alphabets (a, b) denotes significance at *p* < 0.05

To further investigate whether the JAK2/STAT3 pathway mediates the protective effects of ARG, a widely accepted JAK2 inhibitor AG490 was employed. ARG treatment or AG490 treatment both significantly inhibited the phosphorylation level of p-JAK2 and its downstream p-STAT3 induced by FAdV-4 (Fig. 3 A-D) (*P* < *0.05*). And after co-treatment with ARG and AG490, the phosphorylation level of p-JAK2 and p-STAT3 was significantly down-regulated in LMH cells infected with FAdV-4, compared with only ARG treatment or AG490.

In addition, the result showed that the inflammatory cytokines IL-6, IL-1β, and IFN-α that were induced by FAdV-4 were down-regulated in the LMH cell line after treatment with AG490, compared with cells that was infected with FAdV-4 (*P* < *0.05*). After ARG and AG490 co-treatment, the mRNA levels of IL-6, IL-1β and IFN-α were significantly decreased in LMH cells infected with FAdV-4 (*P* < *0.05*), compared with only ARG or AG490 treatment cells (Fig. 3 E–G).

### KEGG functional analysis on the livers infected with FAdV-4

In this study, we concentrated on the pathway about the inflammatory response by the KEGG functional analysis (Ref: 220,192). Table 4 showed that the cytokines pathway and the JAK/STAT pathway were enriched in the liver at post-infection with FAdV-4, compared within mock livers. Furthermore, DEGs including IL-6, IL-1β, IFN-α, JAK and STAT were up-regulated. These reuslts revealed that FAdV-4 infection caused the inflammatory response in livers of broilers, which was regulated by JAK/STAT pathway.

**Table 4:** KEGG analysis on the broiler livers infected with FAdV-4

Pathway	DEGs	Express trend
Cytokines	IL-6、IL-1β、IFN-α	up-regulated
JAK-STAT	JAK、STAT	up-regulated

### FAdV-4 up-regulated the expression of inflammatory cytokines, p-JAK2 and p-STAT3 in broiler liver

**Fig. 4:**
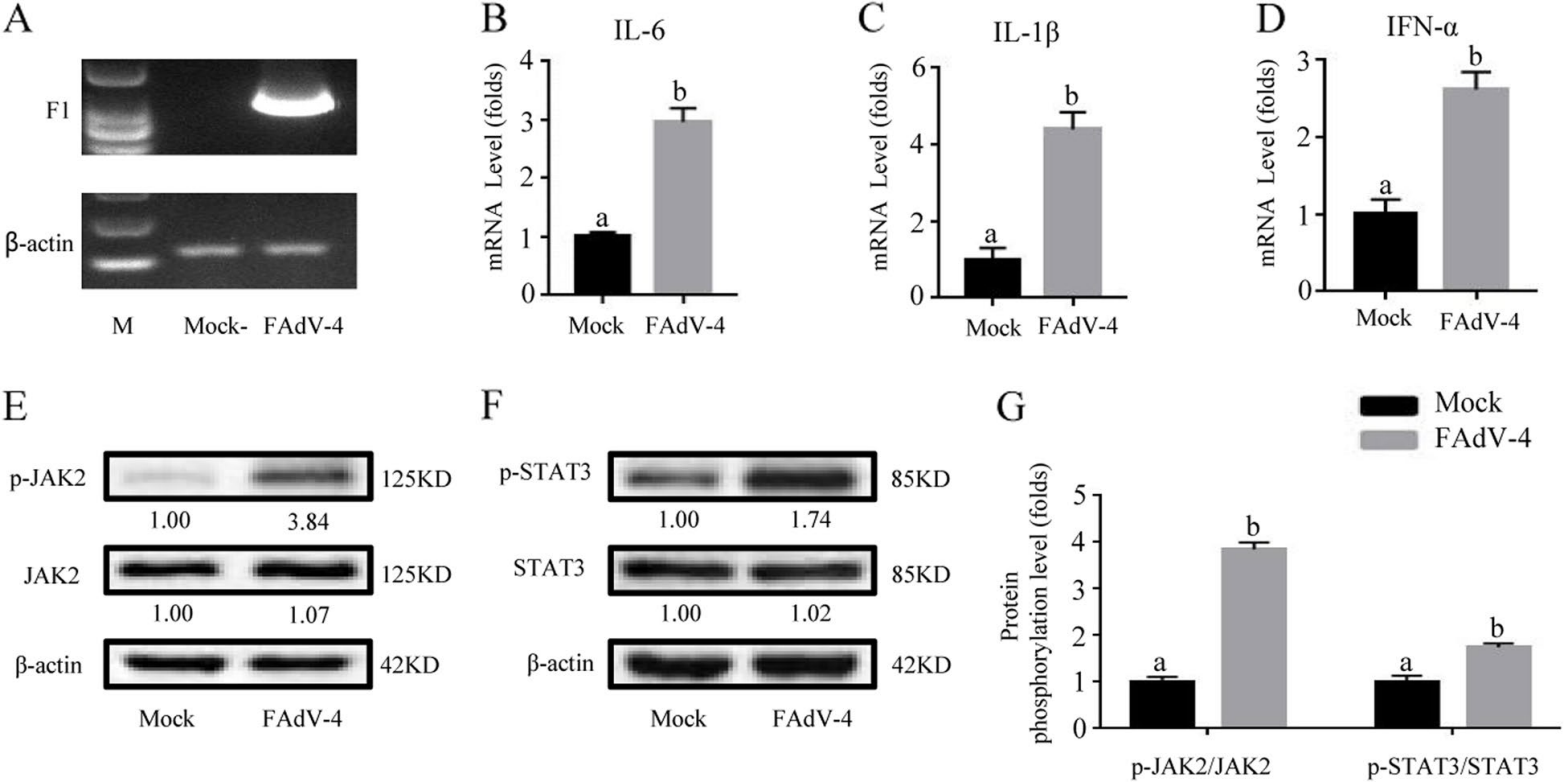
FAdV-4 induced the expression of inflammatory factors and activated the JAK2/STAT3 pathway in the broiler liver. After infection with FAdV-4 24 h (**A**), the virus load of FAdV-4 was determined by PCR with the primers of F1 gene, and the β-actin was used as a conference gene. The mRNA levels of **B** *IL-6*, **C** *IL-1β*, and **D** *IFN-α*, as well as the phosphorylation levels of **E** JAK2 and **F** STAT3, were determined by real-time qPCR and Western blot in the liver of broilers. **G** The degree of protein expression was determined by the depth of WB bands. Histogram is the result of the ratio of the phosphorylated protein to the total protein, and is the digital embodiment of the protein phosphorylation level of JAK2 and STAT3.Significance between the treatments was determined by T-test analysis using SPSS software (Version 20.0). Means with different alphabets (a, b) denotes significance at *p* < 0.05.

Futhermore, we determined the mRNA levels of IL-6, IL-1β, and IFN-α in the liver of FAdV-4-challenged chickens by qPCR. After FAdV-4 infection, the mRNA levels of IL-6, IL-1β, and IFN-α were significantly increased (*P* < *0.05*) (Fig. 4 A-D). Concurrently, as shown in Fig. 4 E–F, the phosphorylation blotting of p-JAK2 and p-STAT3 were significantly improved in livers of FAdV-4 infection broilers (*P* < *0.05*). Histogram results of gray values further confirmed the promotion of FAdV-4 on JAK2 and STAT3 phosphorylation in broilers (Fig. 4 G). These results further supported the KEGG data in vivo.

### Arginine down-regulated the expression of inflammatory cytokines and inhibits JAK2/STAT3 signaling pathway in broiler liver-infected with FAdV-4

**Fig. 5:**
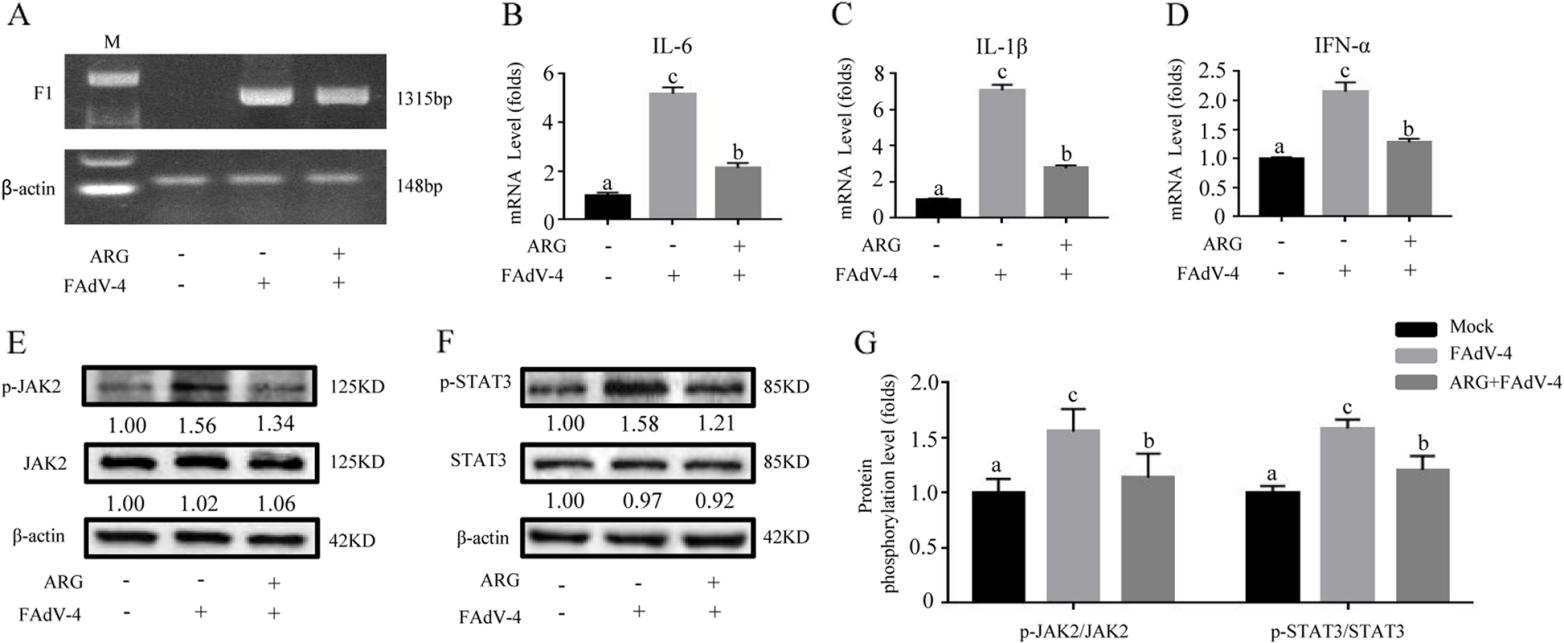
Dietary 1.92% levels of arginine in broilers attenuated inflammatory response and inhibited JAK2/STAT3 signal transduction. Broilers were randomly separated into three experimental groups: Mock and FAdV-4 groups were given a standard diet; ARG + FAdV-4 group, given 1.92% of the arginine level standard diet. At 21 d, FAdV-4 and ARG + FAdV-4 groups were inoculated with FAdV-4, while the mock group was injected with normal saline. After infection with FAdV-4 24 h (**A**), the virus load of FAdV-4 was determined by PCR with the primers of F1 gene, and the β-actin was used as a conference gene. The mRNA levels of **B** *IL-6*, **C** *IL-1β*, and **D** *IFN-α*, as well as the phosphorylation levels of **E** JAK2 and **F** STAT3, were measured and analyzed. **G** The degree of protein expression was determined by the depth of WB bands. Histogram is the result of the ratio of the phosphorylated protein to the total protein, and is the digital embodiment of the protein phosphorylation level of JAK2 and STAT3.Significance between the treatments was determined by T-test analysis using SPSS software (Version 20.0). Means with different alphabets (a, b) denotes significance at *p* < 0.05

In our present study, 1.92% level of dietary arginine treatment could significantly down-regulate the expression of pro-inflammatory factors IL-6, IL-1β, and IFN-α in broiler liver-infected with FAdV-4 (*P* < *0.05*) (Fig. 5 A-D). Moreover, the protein levels of p-JAK2 and p-STAT3 were significantly reduced in 1.92% level of dietary arginine treatment group-infected with FAdV-4 (*P* < *0.05*) (Fig. 5 E–G)*.* These results demonstrated that arginine could down-regulate inflammation response and JAK2/STAT3 pathway in the broiler liver-infected with FAdV-4.

Therefore, these results revealed that arginine alleviated inflammation- induced by FAdV-4 through regulating the JAK2/STAT3 signaling pathway.

## Discussion

The FAdV-4 infection caused severe economic lose to the global poultry industry [28]. At present, the pathogenic mechanism of FAdV-4 infection still has not been fully understood. It was well known that IBH and HHS were typical fatal infectious diseases caused by FAdV-4 [29]. Commonly, the pathological feature of FAdV-4-induced HHS disease were severe hydropericardium and liver inflammation [30]. The major findings were gross lesions in the hydropericardium with the accumulation of yellow gelatinous fluid in the pericardium [31]. At histopathological analysis, the most consistent findings were multifocal areas of necrosis and mononuclear cell infiltration in the liver, including basophilic intranuclear inclusion bodies in hepatocytes [32]. Poultry-infected with FAdV-4 showed obvious multifocal areas of necrosis in the liver and abundant inflammatory cells in liver tissue [33]. Previous studies have shown that the JAK/STAT signaling pathway was a major mechanism of broad cytokine signaling that regulates cell proliferation, growth, apoptosis, and inflammatory responses [34, 35]. Studies on severe acute pancreatitis (SAP) in rats have found that JAK2 and STAT3 protein expression levels were significantly increased following induction of SAP [36]. In our study, results revealed that FAdV-4 infection can activate JAK2/STAT3 signal transduction and induce the expression of inflammatory factors, leading to liver inflammation and hepatic necrosis.

The prevention and control of FAdV-4 infection, especially in response to inflammation, was still not very successful [37]. Recent studies found that arginine regulated the immune system, specifically reducing inflammation [38]. Jiang found that when arginine was added both to the diet of Jian carp or a primary enterocyte culture media, the inflammation induced by lipopolysaccharide was significantly suppressed [39]. Our previous study revealed that FAdV-4 infection disturbed arginine metabolism to benefit its replication [17], and the addition of dietary arginine inhibited FAdV-4 replication [26]. In this present study, we further revealed that arginine treatment significantly inhibited the mRNA levels of IL-6, IL-1β and IFN-α in the broiler livers and LMH cells induced by FAdV-4, respectively.

The JAK/STAT was a classic familial signaling pathway in which each molecule has many subtypes. At the same time, each subtype has different modes of action and phosphorylation sites, which could regulate the different biological properties of cells [40]. In particular, the JAK2/STAT3 pathway has been extensively studied for its inflammatory regulation [12]. Specifically, JAK2 was important for cytokine receptor signaling. Upon activation, JAK2 kinase phosphorylates STAT3, causing inflammation [40, 41]. Evidence suggested that blocking the JAK2/STAT3 signaling transduction could inhibit inflammation [42, 43]. By targeting JAK2/STAT3 signal transduction, it interfered with the phosphorylation of JAK2, a key protein in the pathway, thus blocking the phosphorylation and activity of downstream STAT3 protein, and provided strong protection against cell damage induced by small-molecule substances such as cytokines [42]. Intervention with AG490 (a highly selective JAK2 inhibitor) in a rat model of inflammatory bowel disease (IBD) significantly decreased the levels of IL-6 and IL-17A and increased the levels of IL-10, suggesting a protective effect on IBD [23]. Our present results indicated that arginine could inhibit JAK2/STAT3 signal transduction in inflammatory conditions induced by FAdV-4.

To explore whether JAK2/STAT3 signaling pathway was the leading way for arginine to alleviate FAdV-4-induced inflammation by comparing the inhibition of the JAK2/STAT3 pathway and the expression of inflammatory factors between arginine and AG490 (specific inhibition of JAK2 protein). The results revealed that both arginine and AG490 treatments could reduced inflammation response-induced with FAdV-4. Furthermore, the interesting discovery was that co-treatment with arginine and AG490 further promoted the inhibition of inflammation induced by FAdV-4.

## Conclusion

In summary, arginine down-regulates inflammatory response induced by FAdV-4 via JAK2/STAT3 pathway in vivo and in vitro, which will provide new insight into the prevention against FAdV-4 infection.

## Supplementary Information

**Additional file 1:** **Supplementary Material 1.** Orginal data of WB.

**Additional file 2:** **Supplementary Material 2.** Orginal data of PCR.

## Data Availability

All data generated or analysed during this study are included in this published article [and its supplementary information files].

## Abbreviations

ARG: Arginine
CO_2_: Carbon dioxide
DEGs: Differential expression genes
DMSO: Dimethyl sulfoxide
ECL: Enhanced chemiluminescence
FAdV4: Fowl adenovirus serotype 4
FBS: Fetal bovine serum
HHS: Hepatitis-hydropericardium syndrome
HRP: Horse radish peroxidase
IFN-α: Interferon-α
IL-6: Interleukin-6
IL-1β: Interleukin-1β
JAK: Janus kinase
KEGG: Kyoto encyclopedia of genes and genomes
LMH: Chicken hepatocellular carcinoma cell line
PVDF: Polyvinylidene fluoride
RIPA: Radio immunoprecipitation assay
PBS: Phosphate-buffered saline
PCR: Polymerase chain reaction
qPCR: Real-time fluorescence quantitative polymerase chain reaction
RIPA: Radio-immunoprecipitation assay
SDS-PAGE: Sodium dodecyl sulfate–polyacrylamide gel-electrophoresis
STAT: Signal transducers and activators of transcription
TNF-α: Tumor necrosis factor-alpha
TBST: Tris buffered saline tween
WB: Western blotting

## References

[1] Li R, Li G, Lin J (2018). Fowl adenovirus serotype 4 SD0828 infections causes high mortality rate and cytokine levels in specific pathogen-free chickens compared to ducks. Front Immunol.

[2] Chen Y, Huang R, Qu G (2020). Transcriptome analysis reveals new insight of fowl adenovirus serotype 4 infection. Front Microbiol.

[3] Dhar P, Thakur A, Asrani RK, Katoch V, Sharma M (2018). Protein profile of FAdV-4 based on SDS-PAGE and Western blot isolated from chickens in India. Comp Immunol Microbiol Infect Dis.

[4] Mo KK, Lyu CF, Cao SS (2019). Pathogenicity of an FAdV-4 isolate to chickens and its genomic analysis. J Zhejiang Univ Sci B.

[5] Yu X, Wang Z, Chen H (2018). Serological and pathogenic analyses of fowl adenovirus serotype 4 (FAdV-4) strain in muscovy ducks. Front Microbiol.

[6] Liu YL, Li DF, Gong LM, Yi GF, Gaines AM, Carroll JA (2003). Effects of fish oil supplementation on the performance and the immunological, adrenal, and somatotropic responses of weaned pigs after an Escherichia coli lipopolysaccharide challenge. J Anim Sci.

[7] Wang Z, Zhao J (2019). Pathogenesis of hypervirulent fowl adenovirus serotype 4: the contributions of viral and host factors. Viruses.

[8] Niu Y, Sun Q, Zhang G (2018). Fowl adenovirus serotype 4-induced apoptosis, autophagy, and a severe inflammatory response in liver. Vet Microbiol.

[9] Okugawa S, Ota Y, Kitazawa T (2003). Janus kinase 2 is involved in lipopolysaccharide-induced activation of macrophages. Am J Physiol Cell Physiol.

[10] Siveen KS, Sikka S, Surana R (2014). Targeting the STAT3 signaling pathway in cancer: role of synthetic and natural inhibitors. Biochim Biophys Acta.

[11] Zuo P, Zhu Y, Li Y (2020). Protease-activated receptor 2 deficiency in hematopoietic lineage protects against myocardial infarction through attenuated inflammatory response and fibrosis. Biochem Biophys Res Commun.

[12] Zhang C, Liu J, Yuan C (2019). JAK2/STAT3 is associated with the inflammatory process in periapical granuloma. Int J Clin Exp Pathol.

[13] Gao H, Wu D, Zhang E (2019). Phasic change and apoptosis regulation of JAK2/STAT3 pathway in a type 2 diabetic rat model. Am J Transl Res.

[14] Li M, Zhang X, Wang B (2018). Effect of JAK2/STAT3 signaling pathway on liver injury associated with severe acute pancreatitis in rats. Exp Ther Med.

[15] Qi C, Wang X, Han F (2019). Arginine supplementation improves growth, antioxidant capacity, immunity and disease resistance of juvenile Chinese mitten crab. Eriocheir sinensis Fish Shellfish Immunol.

[16] Zhang B, Li G, Shahid MS (2020). Dietary L-arginine supplementation ameliorates inflammatory response and alters gut microbiota composition in broiler chickens infected with Salmonella enterica serovar Typhimurium. Poult Sci.

[17] Lin Z, Huang R, Zhou J (2020). Fowl Adenovirus Serotype 4 Influences Arginine Metabolism to Benefit Replication. Avian Dis.

[18] Ding LY, Wang YF, Shen YZ (2020). Effects of intravenous arginine infusion on inflammation and metabolic indices of dairy cows in early lactation. Animal.

[19] Krzystek-Korpacka M, Fleszar MG, Bednarz-Misa I (2020). Transcriptional and metabolomic analysis of L-arginine/nitric oxide pathway in inflammatory bowel disease and its association with local inflammatory and angiogenic response: preliminary findings. Int J Mol Sci.

[20] Lan J, Dou X, Li J (2020). L-arginine ameliorates lipopolysaccharide-induced intestinal inflammation through inhibiting the TLR4/NF-κB and MAPK pathways and stimulating β-defensin expression in vivo and in vitro. J Agric Food Chem.

[21] Kawaguchi T, Nomura K, Hirayama Y, Kitagawa T (1987). Establishment and characterization of a chicken hepatocellular carcinoma cell line. LMH Cancer Res.

[22] Gabis KK, Gildemeister OS, Pepe JA, Lambrecht RW, Bonkovsky HL (1996). Induction of heme oxygenase-1 in LMH cells. Comparison of LMH cells to primary cultures of chick embryo liver cells. Biochim Biophys Acta..

[23] Guo J, Wang LY, Wu J, Xu LF, Sun M (2020). The JAK2 inhibitor AG490 regulates the Treg/Th17 balance and alleviates DSS-induced intestinal damage in IBD rats. Clin Exp Pharmacol Physiol.

[24] Gabrashanska M, Teodorova SE, Galvez-Morros M, Mitov M (2004). Effects of glycine-metal compounds on Ascaridia galli-infected chickens expressed by a kinetic model. J Helminthol.

[25] Committee on Nutrient Requirements of Poultry, and National Research Council (1994). Nutrient requirements of poultry.

[26] Lin Z, Zhou J, Chen L, Gao Y, Wang Q, Wang C (2020). Effects of dietary arginine level on immune function and anti-FAdV-4 in broilers. Acta Veterinaria et Zootechnica Sinica.

[27] Wang Q, Huang WR, Chih WY (2019). Cdc20 and molecular chaperone CCT2 and CCT5 are required for the Muscovy duck reovirus p10.8-induced cell cycle arrest and apoptosis. Vet Microbiol.

[28] Hou Y, Mao Z, Wei X (2009). Effects of transforming growth factor-beta1 and vascular endothelial growth factor 165 gene transfer on Achilles tendon healing. Matrix Biol.

[29] Yu L, Liu Z, He W (2020). Hydroxysafflor yellow a confers neuroprotection from focal cerebral ischemia by modulating the crosstalk between JAK2/STAT3 and SOCS3 signaling pathways. Cell Mol Neurobiol.

[30] Li G, Yu G, Niu Y, Cai Y, Liu S (2019). Airborne transmission of a serotype 4 fowl adenovirus in chickens. Viruses.

[31] Schachner A, Marek A, Jaskulska B, Bilic I, Hess M (2014). Recombinant FAdV-4 fiber-2 protein protects chickens against hepatitis-hydropericardium syndrome (HHS). Vaccine.

[32] Li PH, Zheng PP, Zhang TF, Wen GY, Shao HB, Luo QP (2017). Fowl adenovirus serotype 4: epidemiology, pathogenesis, diagnostic detection, and vaccine strategies. Poult Sci.

[33] Ren G, Wang H, Yan Y, Liu F, Huang M, Chen R (2019). Pathogenicity of a fowl adenovirus serotype 4 isolated from chickens associated with hydropericardium-hepatitis syndrome in China. Poult Sci.

[34] Grgić H, Poljak Z, Sharif S, Nagy É (2013). Pathogenicity and cytokine gene expression pattern of a serotype 4 fowl adenovirus isolate. PLoS ONE.

[35] Ashrafizadeh M, Rafiei H, Mohammadinejad R, Afshar EG, Farkhondeh T, Samarghandian S (2020). Potential therapeutic effects of curcumin mediated by JAK/STAT signaling pathway: a review. Phytother Res.

[36] Lu Z, Xiong W, Xiao S (2020). Huanglian Jiedu Decoction ameliorates DSS-induced colitis in mice via the JAK2/STAT3 signalling pathway. Chin Med.

[37] He Z, Chen X, Fu M (2019). Inhibition of fowl adenovirus serotype 4 replication in Leghorn male hepatoma cells by SP600125 via blocking JNK MAPK pathway. Vet Microbiol.

[38] Burin JAM, Fernandes NLM, Snak A, Fireman A, Horn D, Fernandes JIM (2019). Arginine and manganese supplementation on the immune competence of broilers immune stimulated with vaccine against Salmonella Enteritidis. Poult Sci.

[39] Jiang J, Shi D, Zhou XQ (2015). In vitro and in vivo protective effect of arginine against lipopolysaccharide induced inflammatory response in the intestine of juvenile Jian carp (Cyprinus carpio var. Jian). Fish Shellfish Immunol.

[40] Hou Y, Wang K, Wan W, Cheng Y, Pu X, Ye X (2018). Resveratrol provides neuroprotection by regulating the JAK2/STAT3/PI3K/AKT/mTOR pathway after stroke in rats. Genes Dis.

[41] Cao F, Tian X, Li Z (2020). Suppression of NLRP3 inflammasome by erythropoietin via the EPOR/JAK2/STAT3 pathway contributes to attenuation of acute lung injury in mice. Front Pharmacol.

[42] Chen J, Zhang W, Xu Q (2018). Ang-(1–7) protects HUVECs from high glucose-induced injury and inflammation via inhibition of the JAK2/STAT3 pathway. Int J Mol Med.

[43] Li L, Sun L, Qiu Y, Zhu W, Hu K, Mao J (2020). Protective effect of stachydrine against cerebral ischemia-reperfusion injury by reducing inflammation and apoptosis through P65 and JAK2/STAT3 signaling pathway. Front Pharmacol.

