# Correction: Arginine regulates inflammation response-induced by Fowl Adenovirus serotype 4 via JAK2/STAT3 pathway

**DOI:** 10.1186/s12917-022-03306-4

**Published:** 2022-06-20

**Authors:** Silin Xiang, Ruiling Huang, Qing He, Lihui Xu, Changkang Wang, Quanxi Wang

**Affiliations:** 1 College of Animal Science (College of Bee Science), Fujian Agriculture and Forestry University, Fuzhou, 350002 P.R. China; 2 Fujian Key Laboratory of Traditional Chinese Veterinary Medicine and Animal Health, Fujian Agriculture and Forestry University, Fuzhou, 350002 P.R. China; 3 University Key Laboratory for Integrated Chinese Traditional and Western Veterinary Medicine and Animal Healthcare in Fujian Province, Fujian Agriculture and Forestry University, Fuzhou, 350002 P.R. China

**Correction: BMC Vet Res 18, 189 (2022)**

**https://doi.org/10.1186/s12917-022-03282-9**

Following publication of the original article [1], the authors found out error on figures. Corrected figures are provided below.

The original article has been corrected.

Figures 1, 2, 3, 4 and 5

**Fig. 1:**
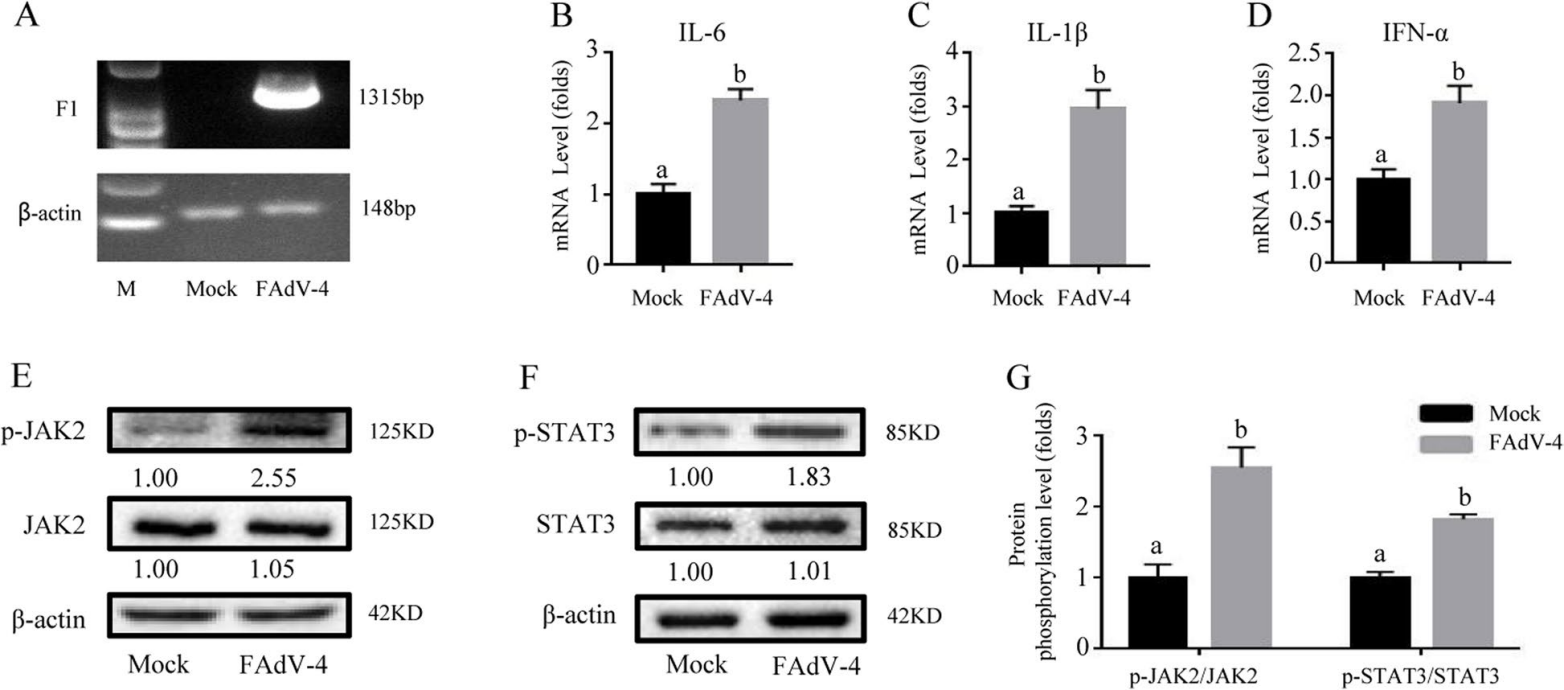
FAdV-4 promoted inflammatory cytokines and the JAK2/STAT3 pathway in LMH cells. The inflammatory indexes of cells infected with or without FAdV-4 were evaluated. After infection with FAdV-4 24 h (**A**), the virus load of FAdV-4 was determined by PCR with the primers of F1 gene, and the β-actin was used as a conference gene. The mRNA levels of cytokines (**B**) *IL-6*, (**C**) *IL-1β*, and (**D**) *IFN-α* was determined by qPCR, while the phosphorylation of crucial proteins (p-JAK2 and p-STAT3) in the JAK2/STAT3 pathway was analyzed by WB (**E-F**). (**G**) The Western blotting was analyzed by gray scale and described by histogram, histogram is the result of the ratio of the phosphorylated protein to the total protein, and is the digital embodiment of the protein phosphorylation level of JAK2 and STAT3. Significance between the treatments was determined by T-test analysis using SPSS software (Version 20.0). Means with different alphabets (a, b) denotes significance at *p* < 0.05. β-actin was used as a control for protein loading, and the following were consistent. All values are expressed as Means ± SD (*n* = 6). The a, b, c, d bars in each panel without a common superscript letter were significantly different (*P* < *0.05*). All remain consistent below unless otherwise stated

**Fig. 2:**
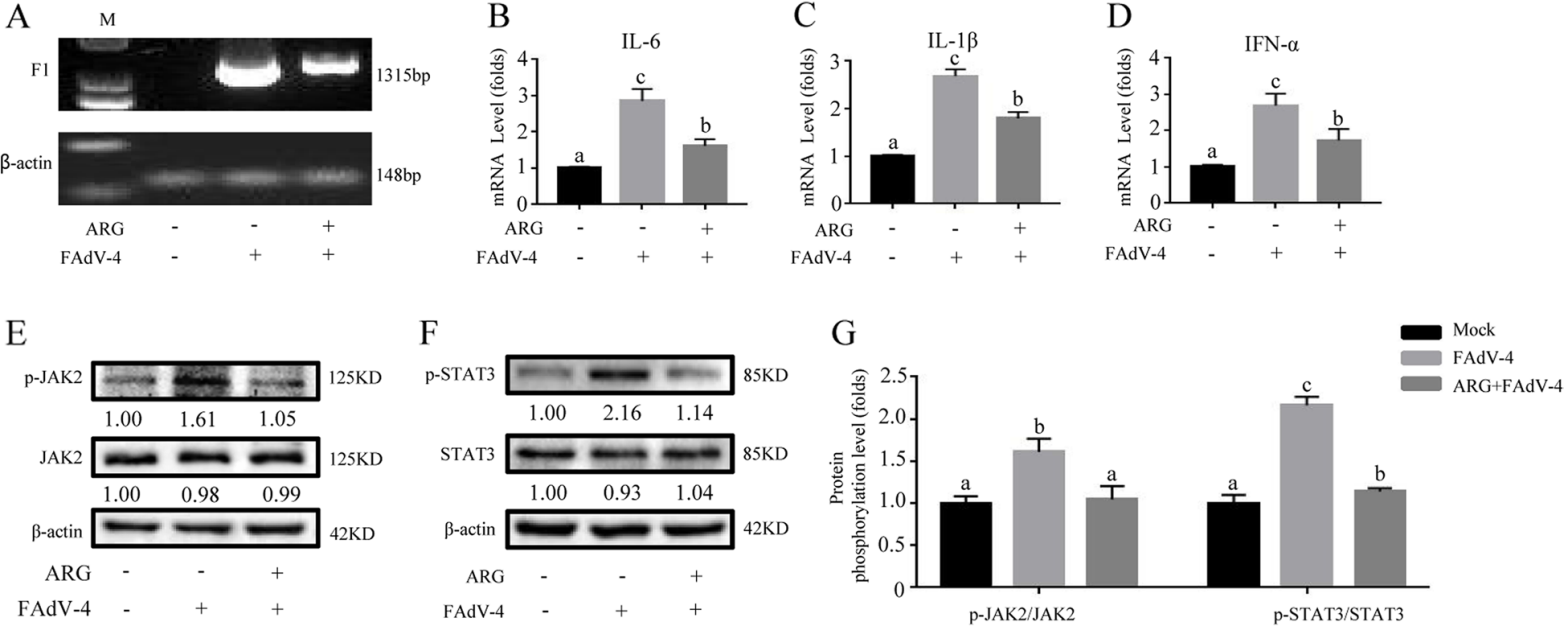
Arginine treatment down-regulated inflammatory response and JAK2/STAT3 pathway-activated by FAdV-4 in LMH cells. The cells were divided into three groups: mock and FAdV-4 groups were cultured with a basal medium containing 10% FBS; ARG + FAdV-4 group was cultured with 300 μg/mL arginine medium. In addition, the FAdV-4 and the ARG + FAdV-4 groups were treated with the FAdV-4 strain NP for 2 h. On the contrary, the mock group was still cultured with a basic medium. After infection with FAdV-4 24 h (**A**), the virus load of FAdV-4 was determined by PCR with the primers of F1 gene, and the β-actin was used as a conference gene. The hepatic mRNA levels of (**B**) *IL-6*, (**C**) *IL-1β*, and (**D**) *IFN-α* were assessed. Similarly, the effect of arginine on the JAK2/STAT3 signaling pathway was determined by evaluating whether arginine interferes with the phosphorylated expression of JAK2 and STAT3 (**E**-**F**). (**G**) The degree of protein expression was determined by the depth of WB bands. Histogram is the result of the ratio of the phosphorylated protein to the total protein, and is the digital embodiment of the protein phosphorylation level of JAK2 and STAT3. Significance between the treatments was determined by T-test analysis using SPSS software (Version 20.0). Means with different alphabets (a, b) denotes significance at *p* < 0.05

**Fig. 3:**
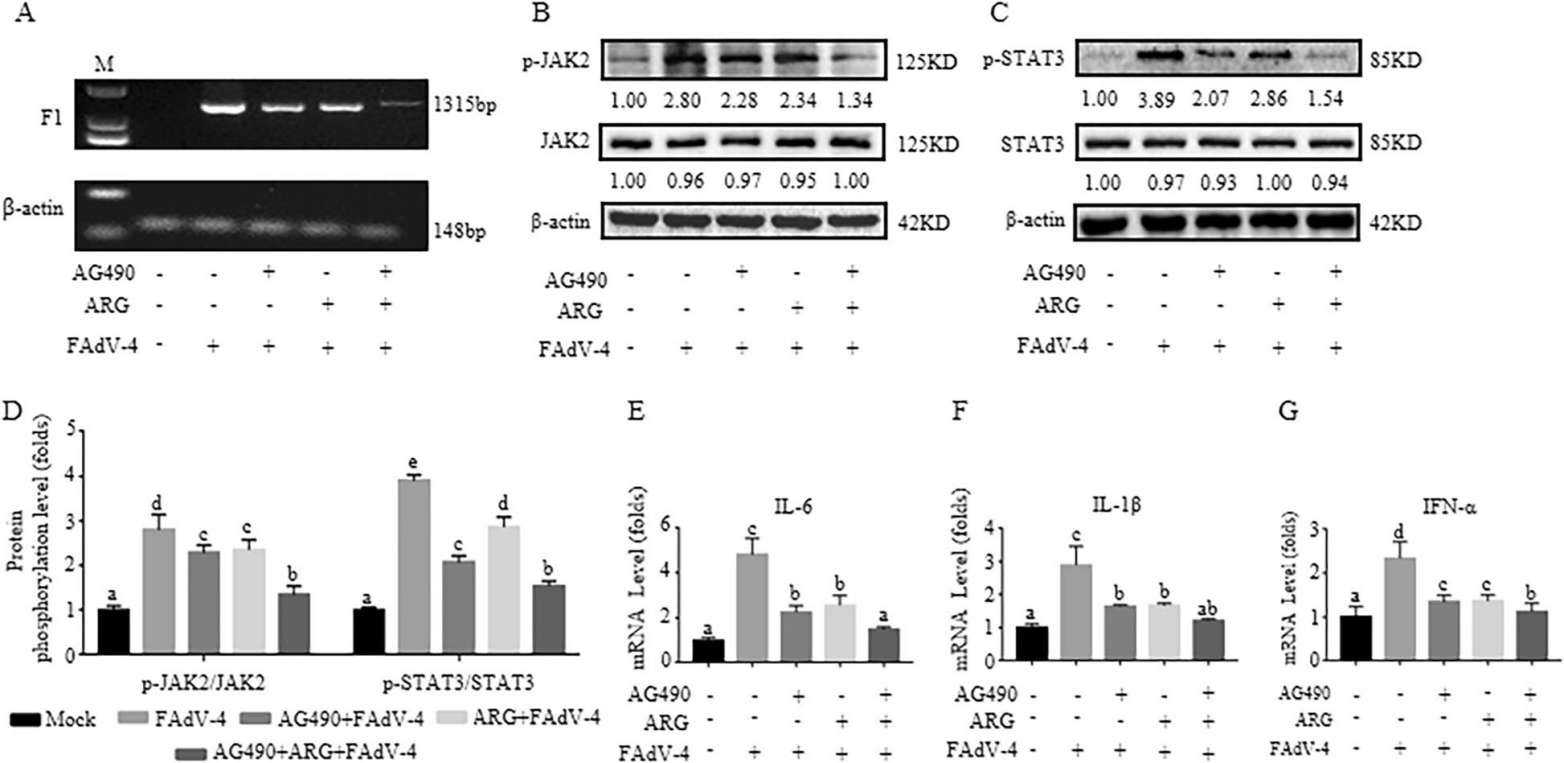
AG490 inhibited the inflammatory effect induced by FAdV-4. To elucidate whether JAK2/STAT3 signaling pathway mediates the effect of arginine in alleviating FAdV-4-induced inflammation, we used AG490, a specific inhibitor of JAK2, in the following trial. LMH cells were treated with 300 μg/mL arginine or 50 nmol/L AG490 and then infected with FAdV-4. After infection with FAdV-4 24 h (**A**), the virus load of FAdV-4 was determined by PCR with the primers of F1 gene, and the β-actin was used as a conference gene. (**B**-**C**) Protein levels of p-JAK2 and p-STAT3 in LMH cells were analyzed by Western blotting and described by histogram, the degree of protein expression was determined by the depth of WB bands. Histogram is the result of the ratio of the phosphorylated protein to the total protein (**D**), and is the digital embodiment of the protein phosphorylation level of JAK2 and STAT3. Meanwhile, the mRNA levels of (**E**) *IL-6*, (**F**) *IL-1β*, and (**G**) *IFN-α* were measured by qPCR. Significance between the treatments was determined by T-test analysis using SPSS software (Version 20.0). Means with different alphabets (a, b) denotes significance at *p* < 0.05

**Fig. 4:**
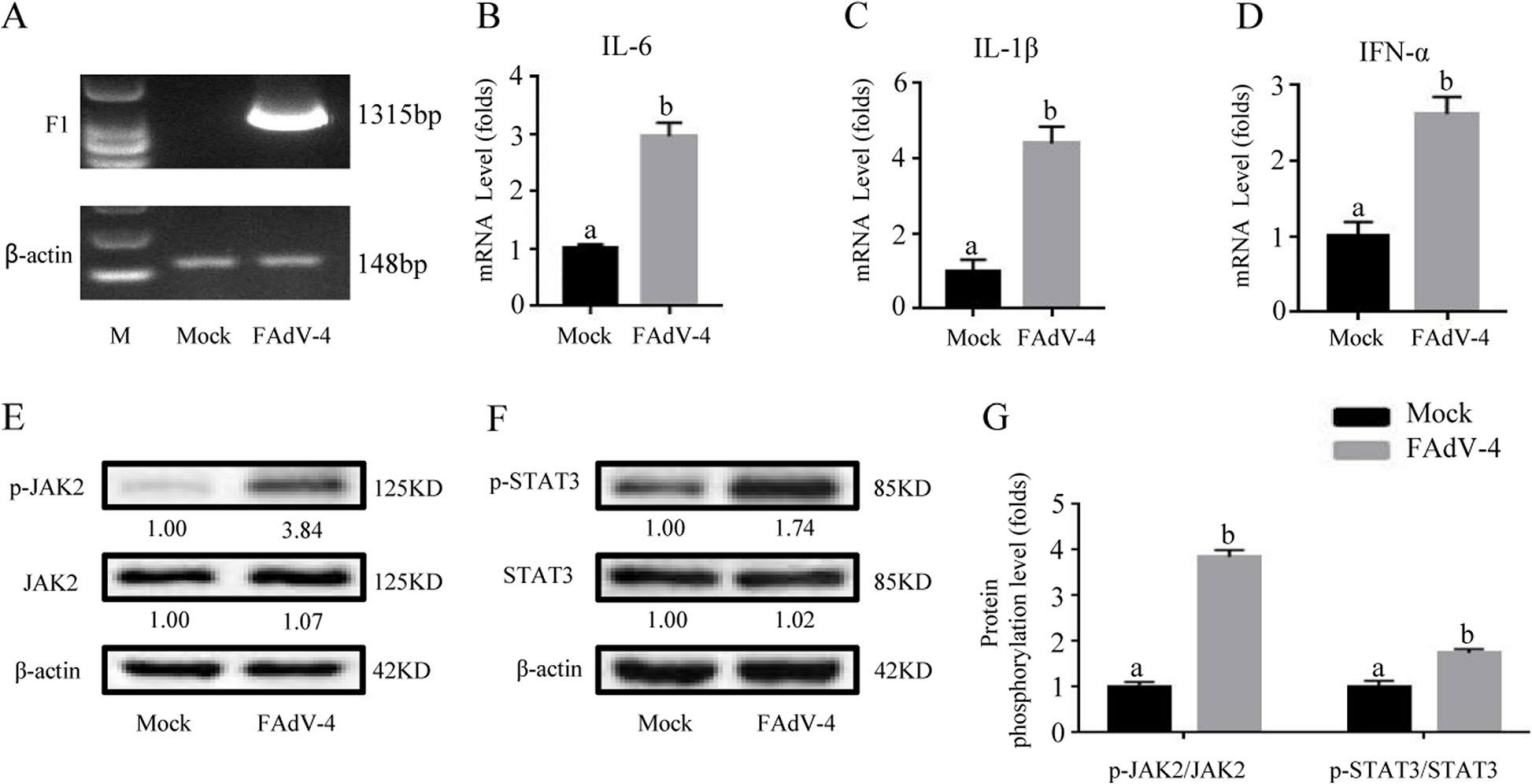
FAdV-4 induced the expression of inflammatory factors and activated the JAK2/STAT3 pathway in the broiler liver. After infection with FAdV-4 24 h (**A**), the virus load of FAdV-4 was determined by PCR with the primers of F1 gene, and the β-actin was used as a conference gene. The mRNA levels of (**B**) *IL-6*, (**C**) *IL-1β*, and (**D**) *IFN-α*, as well as the phosphorylation levels of (**E**) JAK2 and (**F**) STAT3, were determined by real-time qPCR and Western blot in the liver of broilers. (**G**) The degree of protein expression was determined by the depth of WB bands. Histogram is the result of the ratio of the phosphorylated protein to the total protein, and is the digital embodiment of the protein phosphorylation level of JAK2 and STAT3. Significance between the treatments was determined by T-test analysis using SPSS software (Version 20.0). Means with different alphabets (a, b) denotes significance at *p* < 0.05

**Fig. 5:**
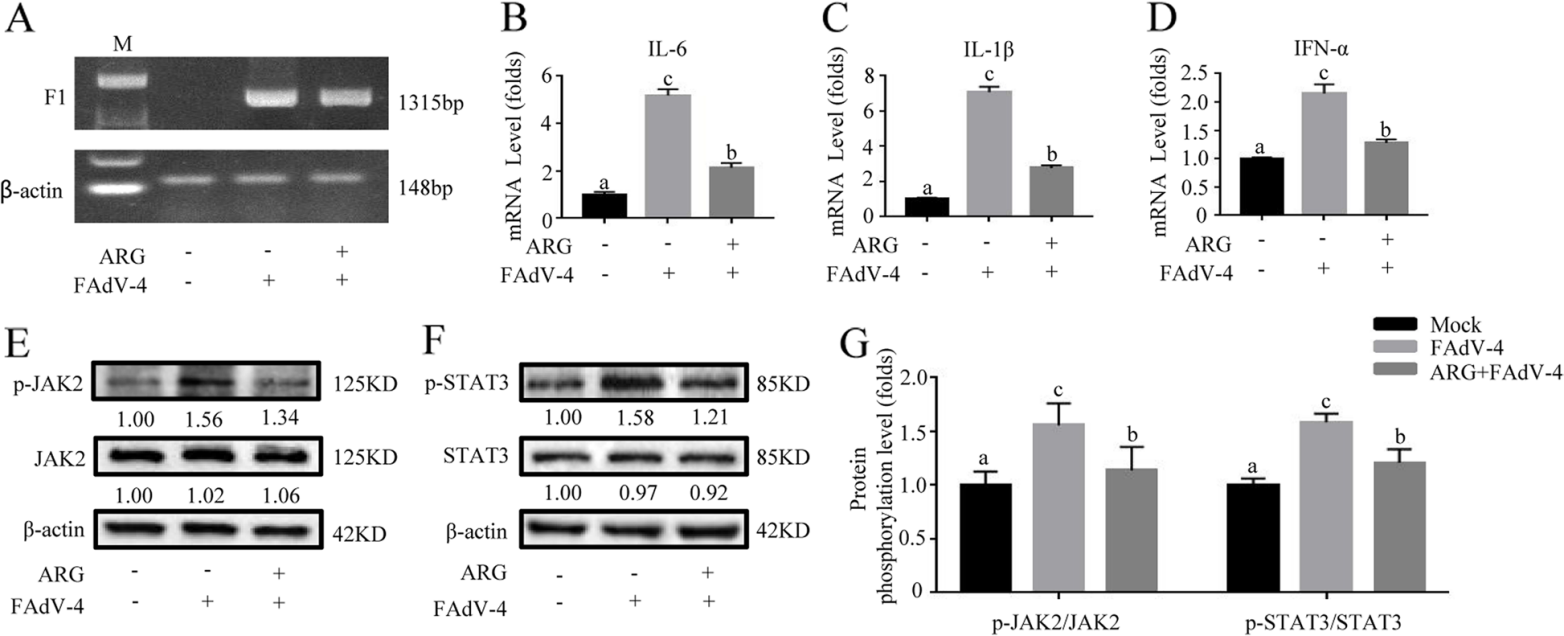
Dietary 1.92% levels of arginine in broilers attenuated inflammatory response and inhibited JAK2/STAT3 signal transduction. Broilers were randomly separated into three experimental groups: Mock and FAdV-4 groups were given a standard diet; ARG + FAdV-4 group, given 1.92% of the arginine level standard diet. At 21 d, FAdV-4 and ARG + FAdV-4 groups were inoculated with FAdV-4, while the mock group was injected with normal saline. After infection with FAdV-4 24 h (**A**), the virus load of FAdV-4 was determined by PCR with the primers of F1 gene, and the β-actin was used as a conference gene. The mRNA levels of (**B**) *IL-6*, (**C**) *IL-1β*, and (**D**) *IFN-α*, as well as the phosphorylation levels of (**E**) JAK2 and (**F**) STAT3, were measured and analyzed. (**G**) The degree of protein expression was determined by the depth of WB bands. Histogram is the result of the ratio of the phosphorylated protein to the total protein, and is the digital embodiment of the protein phosphorylation level of JAK2 and STAT3. Significance between the treatments was determined by T-test analysis using SPSS software (Version 20.0). Means with different alphabets (a, b) denotes significance at *p* < 0.05
