# Supplementary material for: Arginine regulates inflammation response-induced by Fowl Adenovirus serotype 4 via JAK2/STAT3 pathway
Source: BMC Vet Res. 2022 May 19;18:189. doi: 10.1186/s12917-022-03282-9 (PMC9118595; doi:10.1186/s12917-022-03282-9)

Uncropped Western blot image in Fig. 1

Fig. 1E p-JAK2

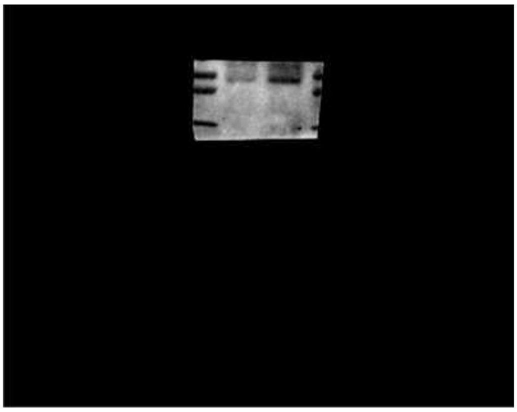

Fig. 1E JAK2

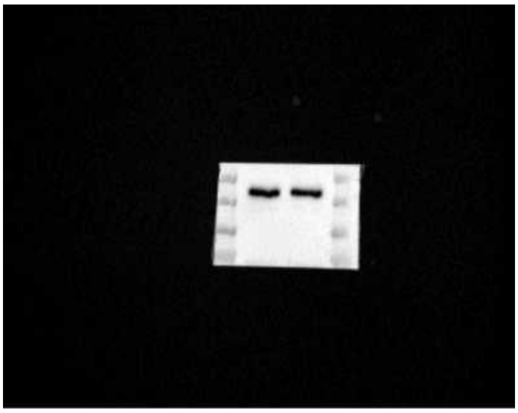

Fig. 1E β-actin

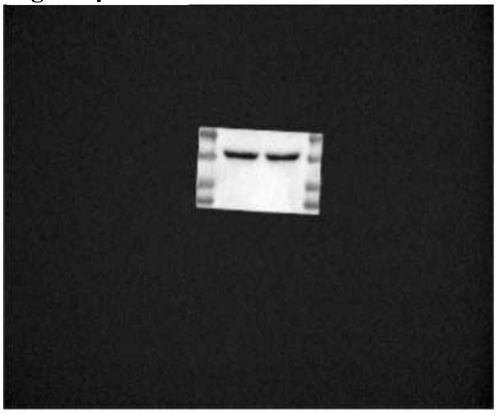

Fig. 1 F STAT3

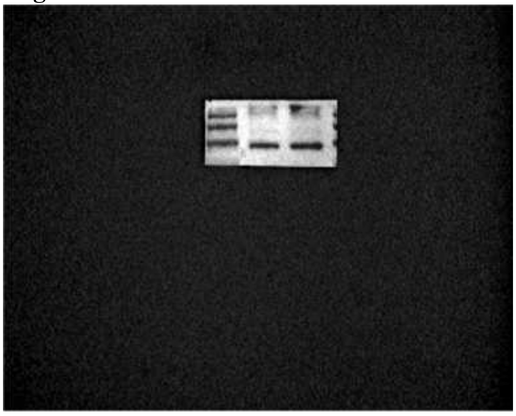

Fig. 1F p-STAT3

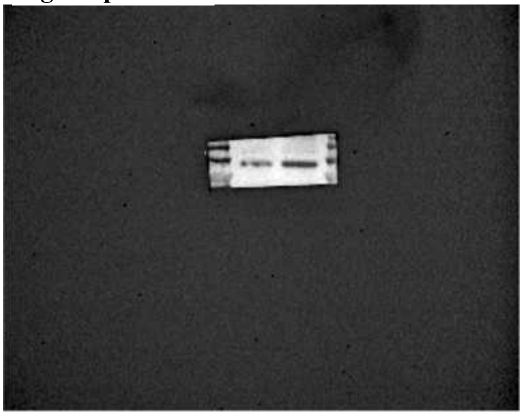

Fig. 1F β-actin

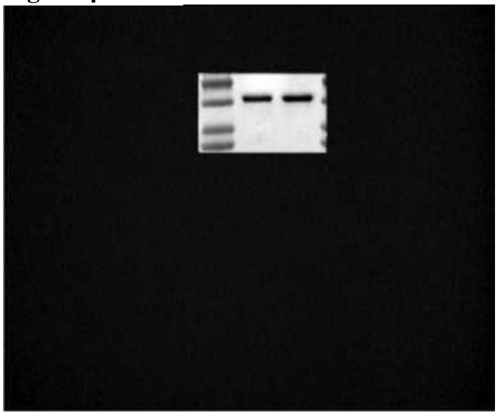

Uncropped Western blot image in Fig. 2

Fig. 2E p-JAK2

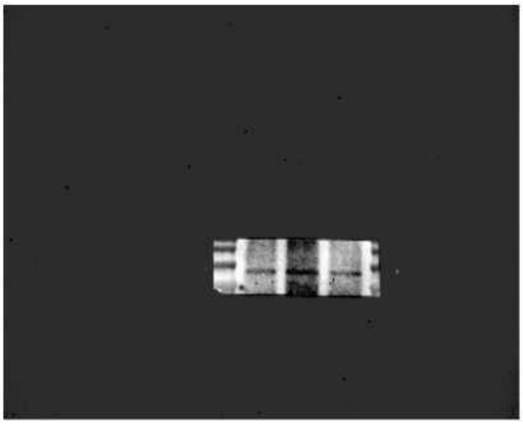

Fig. 2E JAK2

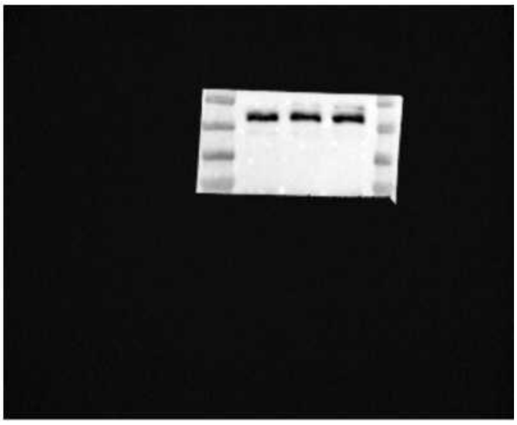

Fig. 2E β-actin

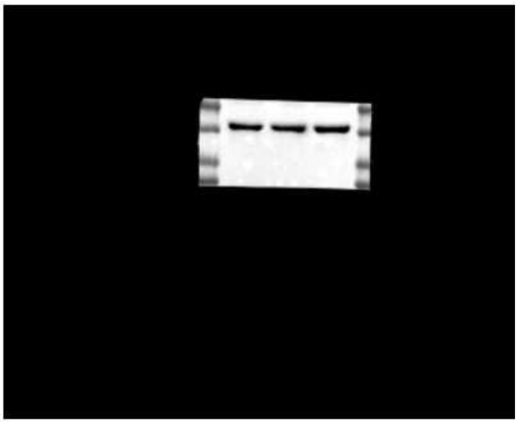

Fig. 2F p-STAT3

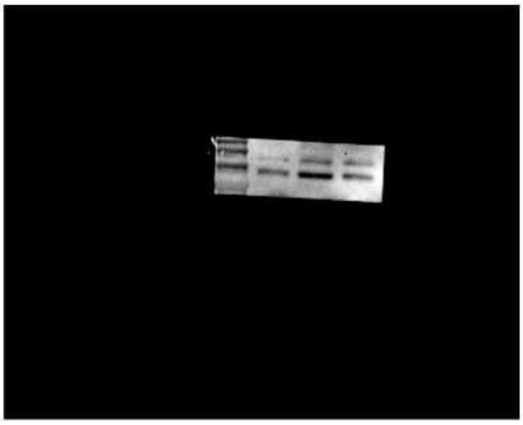

Fig. 2F STAT3

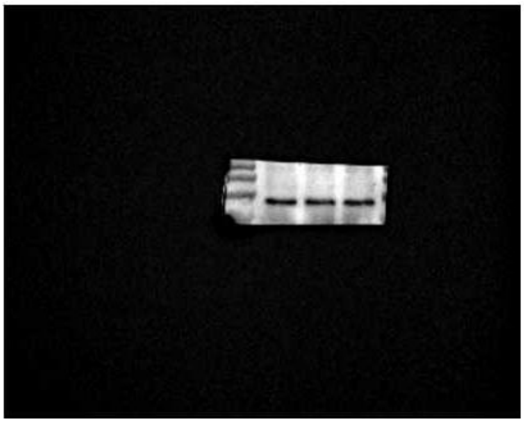

Fig. 2F β-actin

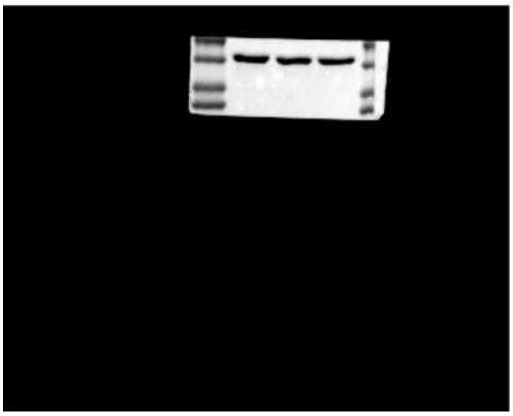

Uncropped Western blot images in Fig. 3

Fig. 3B p-JAK2

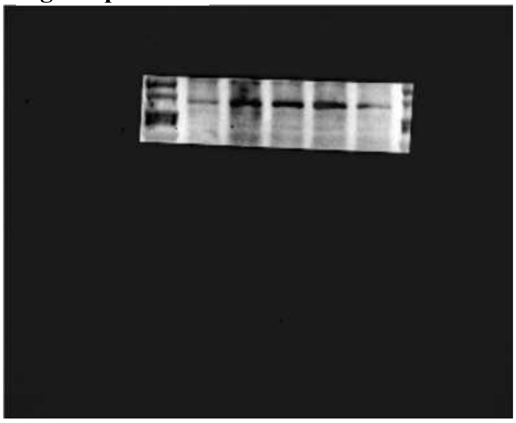

Fig. 3B JAK2

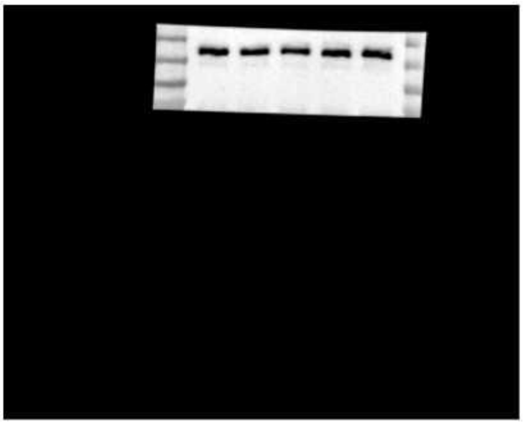

Fig. 3B β-actin

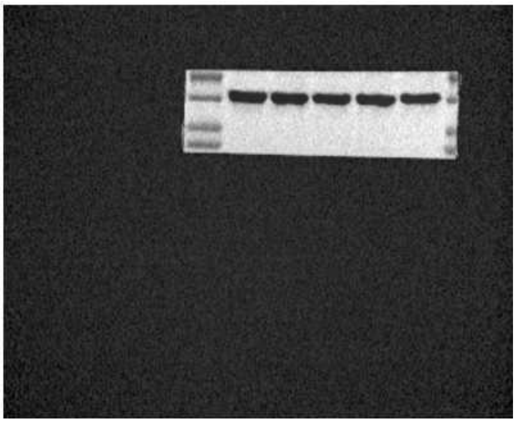

Fig. 3C p-STAT3

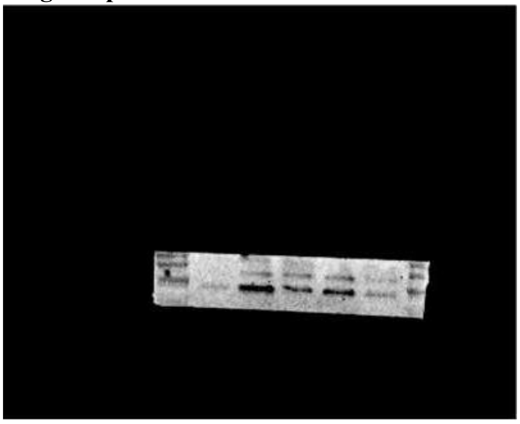

Fig. 3C STAT3

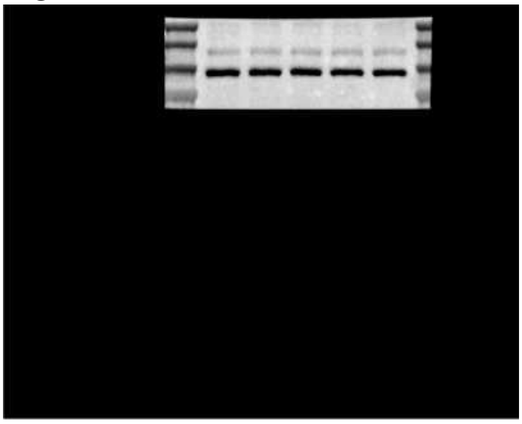

Fig. 3C β-actin

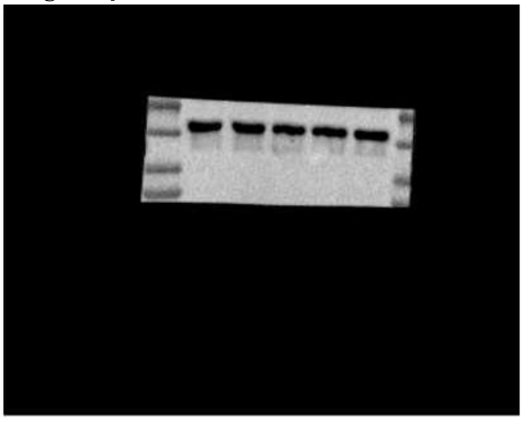

Uncropped Western blot images in Fig.6

Fig.6 E p-JAK2

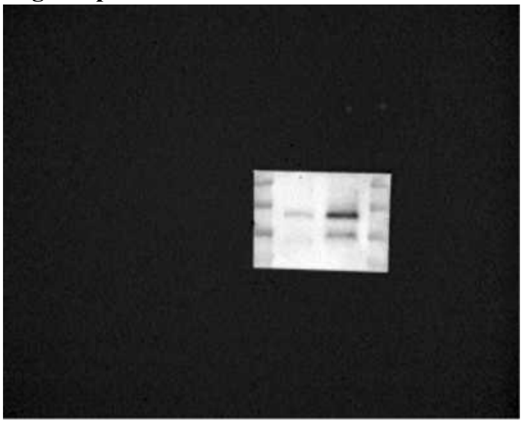

Fig.6E JAK2

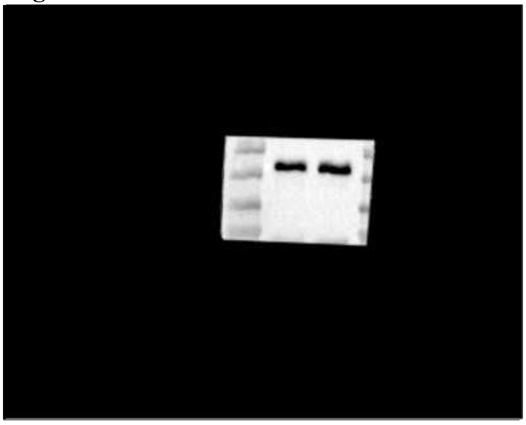

Fig. 6E β-actin

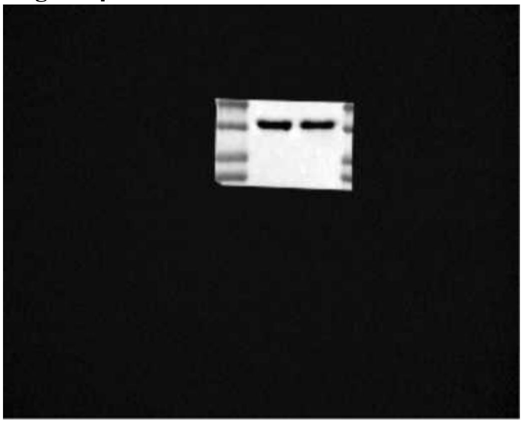

Fig. 6F p-STAT3

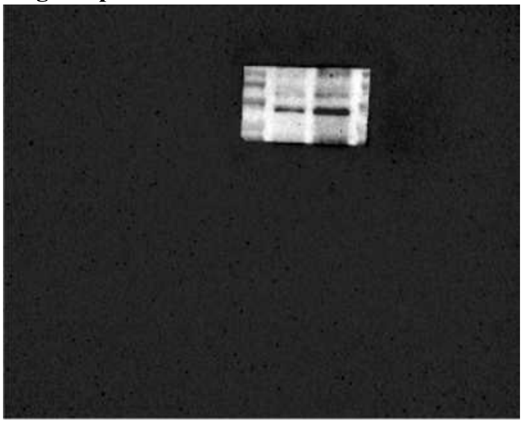

Fig. 6F STAT3

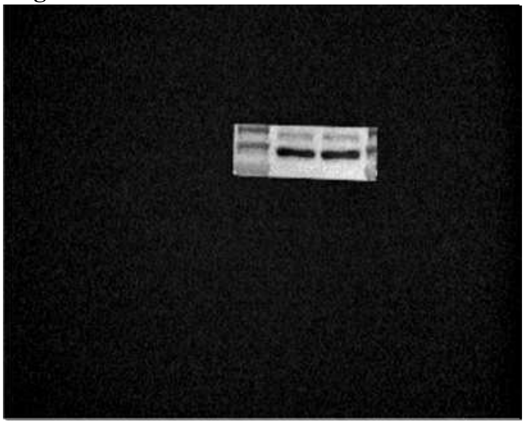

Fig. 6F β-actin

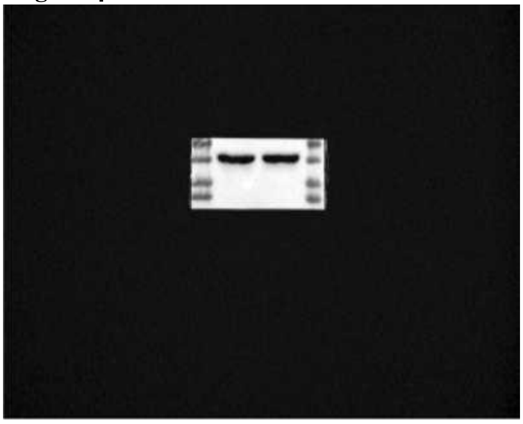

Supplemental Fig 7. Uncropped Western blot images

Fig. 7E p-JAK2

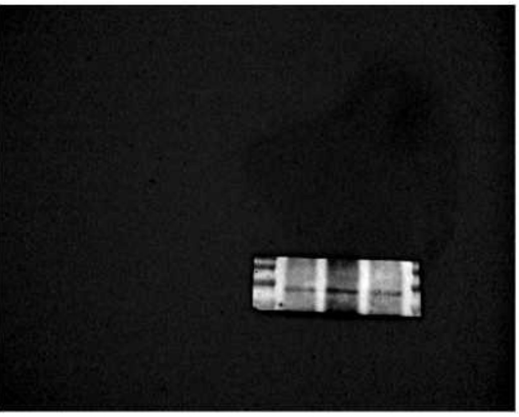

Fig. 7E JAK2

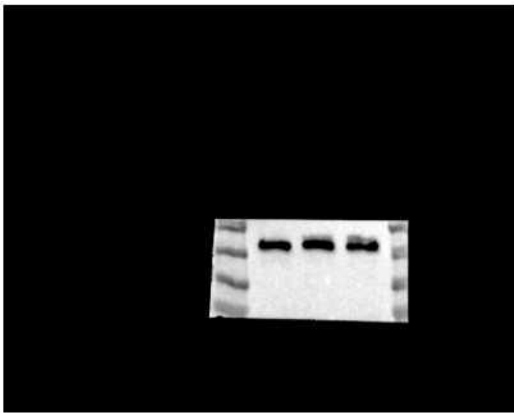

Fig. 7E β-actin

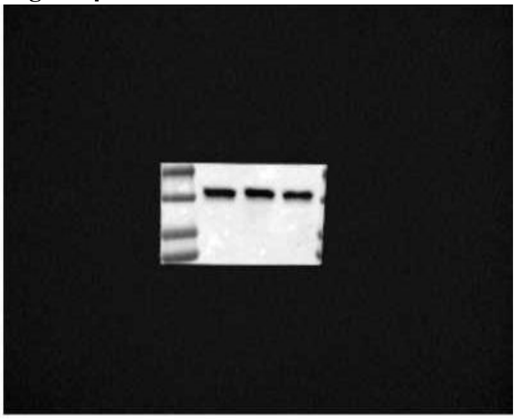

Fig. 7F p-STAT3

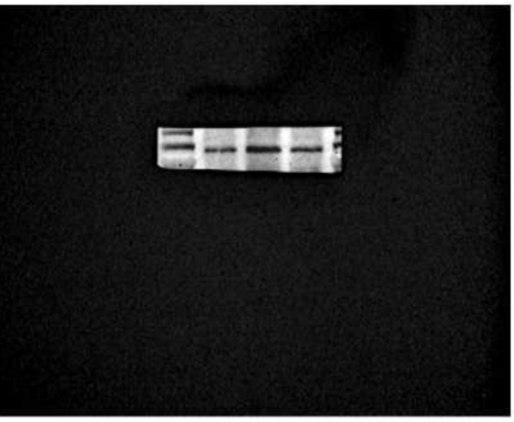

Fig. 7F STAT3

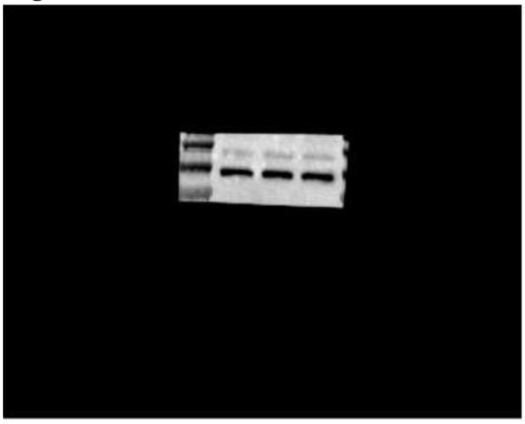

Fig. 7F β-actin

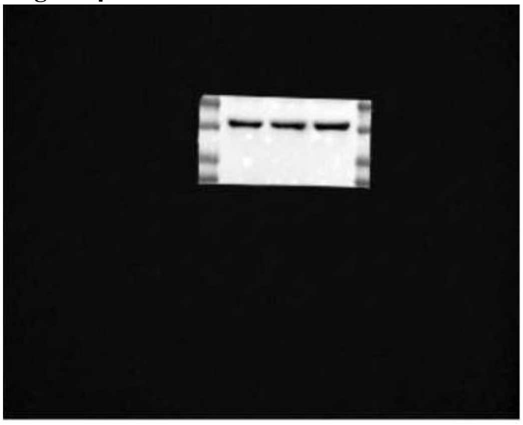

Supplement: Supplementary file 1 — Additional file 1: Supplementary Material 1. Orginal data of WB. [file 12917_2022_3282_MOESM1_ESM.pdf]
