# Supplementary material for: Arginine regulates inflammation response-induced by Fowl Adenovirus serotype 4 via JAK2/STAT3 pathway
Source: BMC Vet Res. 2022 May 19;18:189. doi: 10.1186/s12917-022-03282-9 (PMC9118595; doi:10.1186/s12917-022-03282-9)

Raw Data of PCR in Fig.1

Fig.1A F1

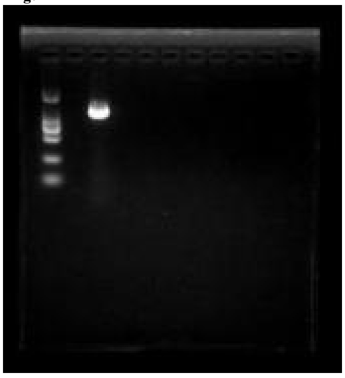

Fig.1A β-actin

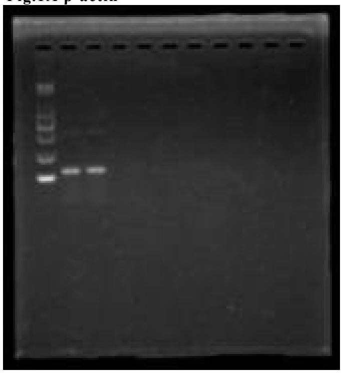

Raw Data of PCR in Fig.2

Fig.2A F1

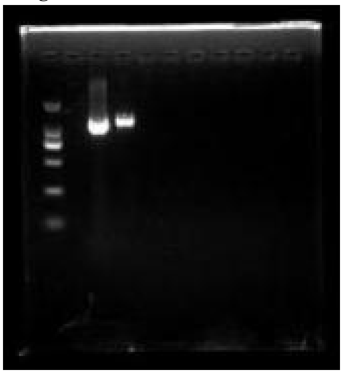

Fig.2A β-actin

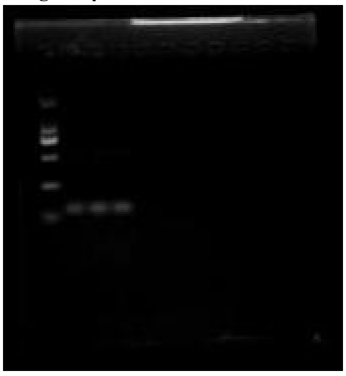

Raw Data of PCR in Fig.3

Fig.3A F1

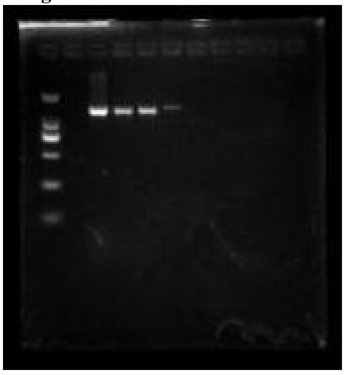

Fig.3A β-actin

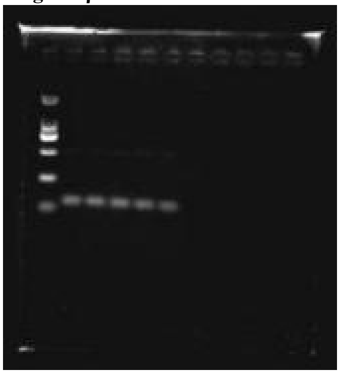

Raw Data of PCR in Fig.6

Fig.6A F1

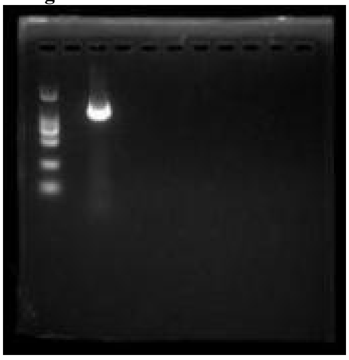

Fig.6A β-actin

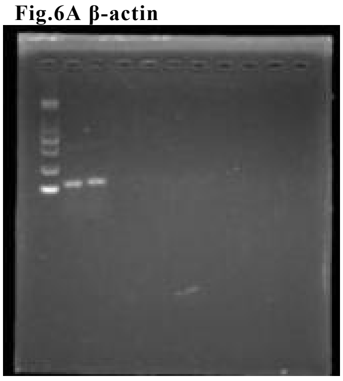

Raw Data of PCR in Fig.7

Fig.7A F1 Fig.7A β-actin

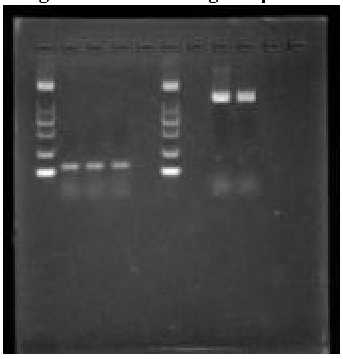

Supplement: Supplementary file 2 — Additional file 2: Supplementary Material 2. Orginal data of PCR. [file 12917_2022_3282_MOESM2_ESM.pdf]
